# Carriage of Extended-Spectrum-Beta-Lactamase- and AmpC Beta-Lactamase-Producing Escherichia coli Strains from Humans and Pets in the Same Households

**DOI:** 10.1128/AEM.01613-20

**Published:** 2020-11-24

**Authors:** Leah J. Toombs-Ruane, Jackie Benschop, Nigel P. French, Patrick J. Biggs, Anne C. Midwinter, Jonathan C. Marshall, Maggie Chan, Dragana Drinković, Ahmed Fayaz, Michael G. Baker, Jeroen Douwes, Mick G. Roberts, Sara A. Burgess

**Affiliations:** a ^*m*^EpiLab, School of Veterinary Science, Massey University, Palmerston North, New Zealand; b School of Fundamental Sciences, Massey University, Palmerston North, New Zealand; c Microbiology Department, North Shore Hospital, Auckland, New Zealand; d Department of Public Health, University of Otago, Wellington, New Zealand; e Centre for Public Health Research, Massey University, Wellington, New Zealand; f School of Natural and Mathematical Sciences, Massey University, Albany, New Zealand; INRS—Institut Armand-Frappier

**Keywords:** ESBL, *Enterobacteriaceae*, *Escherichia coli*, antibiotic resistance

## Abstract

Extended-spectrum-beta-lactamase (ESBL)- or AmpC beta-lactamase (ACBL)-producing Escherichia coli bacteria are the most common cause of community-acquired multidrug-resistant urinary tract infections (UTIs) in New Zealand. The carriage of antimicrobial-resistant bacteria has been found in both people and pets from the same household; thus, the home environment may be a place where antimicrobial-resistant bacteria are shared between humans and pets. In this study, we sought to determine whether members (pets and people) of the households of human index cases with a UTI caused by an ESBL- or ACBL-producing E. coli strain also carried an ESBL- or ACBL-producing *Enterobacteriaceae* strain and, if so, whether it was a clonal match to the index case clinical strain. Index cases with a community-acquired UTI were recruited based on antimicrobial susceptibility testing of urine isolates. Fecal samples were collected from 18 non-index case people and 36 pets across 27 households. Eleven of the 27 households screened had non-index case household members (8/18 people and 5/36 animals) positive for ESBL- and/or ACBL-producing E. coli strains. Whole-genome sequence analysis of 125 E. coli isolates (including the clinical urine isolates) from these 11 households showed that within seven households, the same strain of ESBL-/ACBL-producing E. coli was cultured from both the index case and another person (5/11 households) or pet dog (2/11 households). These results suggest that transmission within the household may contribute to the community spread of ESBL- or ACBL-producing E. coli.

**IMPORTANCE** *Enterobacteriaceae* that produce extended-spectrum beta-lactamases (ESBLs) and AmpC beta-lactamases (ACBLs) are important pathogens and can cause community-acquired illnesses, such as urinary tract infections (UTIs). Fecal carriage of these resistant bacteria by companion animals may pose a risk for transmission to humans. Our work evaluated the sharing of ESBL- and ACBL-producing E. coli isolates between humans and companion animals. We found that in some households, dogs carried the same strain of ESBL-producing E. coli as the household member with a UTI. This suggests that transmission events between humans and animals (or vice versa) are likely occurring within the home environment and, therefore, the community as a whole. This is significant from a health perspective, when considering measures to minimize community transmission, and highlights that in order to manage community spread, we need to consider interventions at the household level.

## Abstract (precis)

*Enterobacteriaceae* that produce extended-spectrum beta-lactamases (ESBLs) and AmpC beta-lactamases (ACBLs) are important pathogens and can cause community-acquired illnesses, such as urinary tract infections (UTIs). Fecal carriage of these resistant bacteria by companion animals may pose a risk for transmission to humans. Our work evaluated the sharing of ESBL- and ACBL-producing E. coli isolates between humans and companion animals. We found that in some households, dogs carried the same strain of ESBL-producing E. coli as the household member with a UTI. This suggests that transmission events between humans and animals (or vice versa) are likely occurring within the home environment and, therefore, the community as a whole. This is significant from a health perspective, when considering measures to minimize community transmission, and highlights that in order to manage community spread, we need to consider interventions at the household level.

## INTRODUCTION

Escherichia coli is a commensal microorganism found both inside and outside the mammalian large intestine and is commonly used as an indicator of fecal contamination (1). E. coli can cause disease and is responsible for most community-acquired (as opposed to hospital-acquired) urinary tract infections (UTIs) (2). Treatment can be complicated by antimicrobial resistance (3), particularly against extended-spectrum cephalosporins (4). Increasingly, enzymes, such as AmpC beta-lactamases (ACBLs) and extended-spectrum beta-lactamases (ESBLs), have been reported in pathogenic bacteria, including E. coli (3). Resistance to beta-lactams is also associated with multidrug resistance (5).

ESBL- and AmpC-producing *Enterobacteriaceae*, predominantly E. coli and, to a lesser extent, Klebsiella pneumoniae, are the most common cause of multidrug-resistant (MDR) UTIs in the New Zealand community (6). Surveillance from 2011 to 2016 showed an increasing infection rate in New Zealand, with a national period prevalence rate of 11.1 per 100,000 people for ESBL-producing *Enterobacteriaceae* from a clinical sample in 2016 (7). In contrast to human infections, antimicrobial resistance surveillance data are lacking for clinical isolates from animals in New Zealand, although cephalosporin resistance (including ESBL and AmpC production) has been found in bacterial isolates from companion animals (8). The transmission of antimicrobial-resistant (AMR) bacteria between cohabiting animals and humans means that antimicrobial interventions in one individual may indirectly affect the microbiome (and its resistance to antibiotics) of other individuals in the same household. A New Zealand study found that 36 of 83 (43%) patients with a community-acquired UTI associated with plasmid-mediated AmpC beta-lactamase-producing E. coli had not been given any antimicrobial treatment in the previous 6 months, nor had they been hospitalized (9). This would suggest that there are other drivers (besides antimicrobial use) for the selection and spread of MDR bacteria in the community, and close contact with household members, including pets, should be considered a potential source.

The family pet plays a central role in the lives of many people, with some behaviors facilitating the transmission of bacteria between people and their pets (10). In New Zealand, over 60% of households have a companion animal, with this rate of pet ownership being among the highest in the world (11). Pets have been shown to share similar E. coli and K. pneumoniae strains with humans in the same household as well as some of the same clonal lineages associated with UTIs in both humans and pets (12–15). Healthy cats and dogs have also been shown to carry E. coli sequence types (STs) that are usually associated with human extraintestinal infection (16). Sharing of other genetically similar bacteria has been observed between cohabiting pets and people. For example, contemporaneous carriage of clonal methicillin-resistant Staphylococcus aureus was observed in 57% of positive humans and pets sharing the same household environment (17). A similar association was reported for *Staphylococcus* species in other studies (18, 19). Reverse zoonotic transfer has also been implicated in methicillin-resistant S. aureus (MRSA) transfer events (19, 20).

Human-to-human transmission within the household is also an important pathway for the spread of AMR bacteria within the community. Family members can share fecal and oral bacteria (21), potentially resulting in pathogenic bacteria being harbored quiescently by individuals and causing disease in cohabiting persons (or pets) (22, 23). In a country with a relatively low prevalence of carriage of extended-spectrum-beta-lactamase-producing E. coli, such as New Zealand (24), the family may be an important reservoir of antimicrobial-resistant bacteria. Isolation of closely related AMR bacteria from cohabiting people has been demonstrated with MRSA (25, 26). The transmission of ESBL-producing sequence type 131 (ST131) E. coli strains (the leading cause of urinary tract infections) within households has also been observed (27), in addition to the sharing of urinary tract infection-causing E. coli strains between sexual partners (28). There is a paucity of information on the household carriage of ESBL-producing E. coli from countries with high rates of pet ownership, such as New Zealand.

This study aimed to evaluate whether the same strains of ESBL- or ACBL-producing E. coli were shared between humans and pets within households. The study focused on the genomic relatedness of ESBL- or ACBL-producing E. coli isolates obtained from persons with community-acquired UTIs and their household members (people and/or pets).

## RESULTS

### Summary of households.

Seventy-two human index cases (individuals suspected to have had a UTI) were invited to submit fecal samples from themselves, as well as any other members of the household, including cats or dogs, that lived with them in the home. Sixty-seven (93%) of the invited index cases submitted fecal samples, while 27 (27/67; 40%) of these case households submitted samples from other members of the household (including pets).

In these 27 households that submitted fecal samples from multiple household members, the index case person was predominantly female (24/27; 89%), and 12/27 (44%) were over 65 years old. All the index cases reported receiving antimicrobial treatment in the prior 6 months, including treatment for the index UTI. Twenty-three households (23/27; 85%) submitted samples from pets, 15 households (15/27; 55%) submitted fecal samples from other people, and 11 households (11/27; 41%) submitted samples from both pets and other people (see Table S1 in the supplemental material). Fecal samples from these 27 households were collected between December 2015 and January 2017 and were obtained between 31 and 180 days after the case UTI urine sample was provided (median time, 63 days; mean time, 77 days).

### Carriage of ESBL-/ACBL-producing *Enterobacteriaceae* within households.

**FIG 1:**
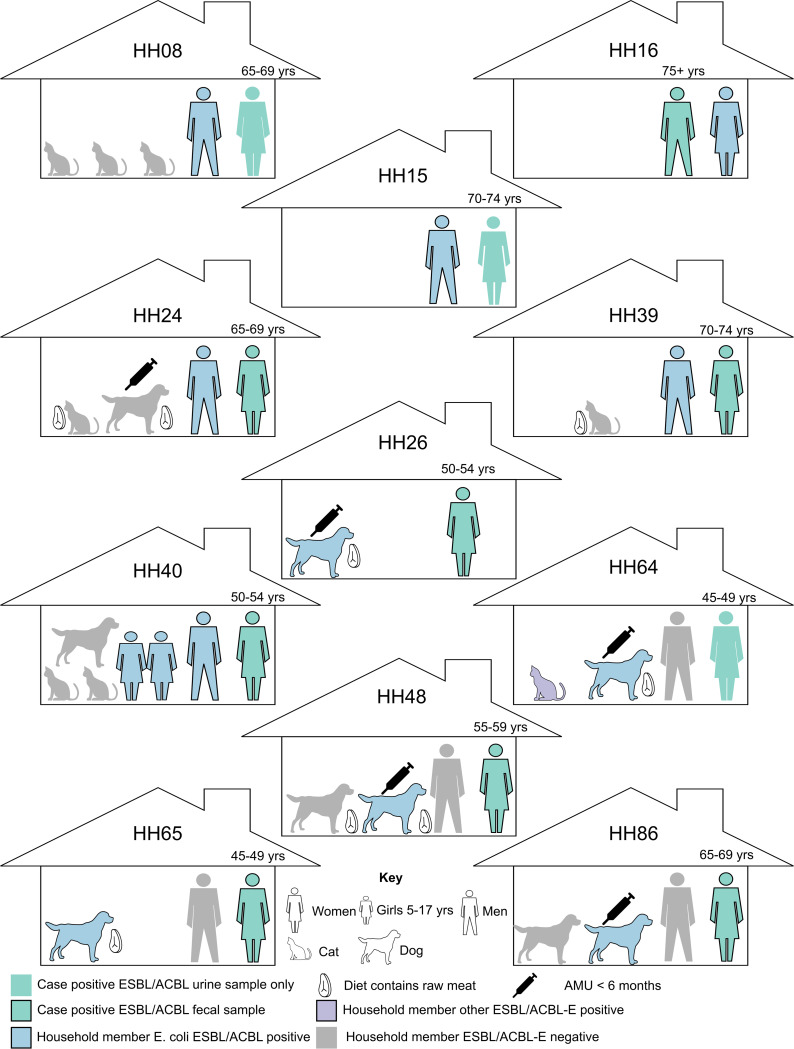
Description of the 11 households where multiple household members were identified to be harboring ESBL-/ACBL-producing E. coli strains and whether household members were positive or negative for the carriage of ESBL- and/or ACBL-producing E. coli. The age of each case participant is marked above the case. ESBL/ACBL-E, ESBL-/ACBL-producing *Enterobacteriaceae*; AMU, antimicrobial use.

Among the 23 households (of the 27 total) that submitted fecal samples from pets, eight (8/23; 35%) households had pets that were positive for an *Enterobacteriaceae* strain with an ESBL-/ACBL-producing phenotype, isolated from nine (6/18 dogs and 3/18 cats) individual animals (Table S1). Five of six positive dogs (5/18; 28% of dogs) were positive for ESBL-/plasmid-mediated ACBL-producing E. coli (from five households). In previous studies, raw meat diets and prior antimicrobial use have been identified as risk factors for ESBL-producing *Enterobacteriaceae* carriage in companion animals (29, 30). Significantly more dogs (15/18; 83%) than cats (4/18; 22%) were reported to eat raw meat as part of their diet (*P* = 0.0006) (Table S1). However, there was no association between the consumption of raw meat and the carriage of ESBL-producing *Enterobacteriaceae* by pets (*P* = 0.23). Antimicrobial treatment was reported in four of the five dogs positive for ESBL-producing E. coli (Fig. 1; Table S1). One dog, with no prior antimicrobial treatment, was positive for other ESBL-/ACBL-producing *Enterobacteriaceae*. There was no association between prior antimicrobial treatment and the presence of ESBL-/ACBL-producing *Enterobacteriaceae* (*P* = 0.23). Significantly more dogs (5/18; 28%) than cats (0/18; 0%) were positive for ESBL-/plasmid-mediated ACBL-producing E. coli (*P = *0.02).

Similarly, among the 15 households that submitted fecal samples from people other than the index case, seven (7/15; 47%) households were positive for ESBL-/ACBL-producing *Enterobacteriaceae*, isolated from nine (9/18; 50%) non-index case individuals. Two of these nine individuals had been treated with antimicrobials in the 6 months prior to the sampling of feces (as reported by the index case). None of the household contacts were reported (by the index case) to have had an MDR infection. In total, 15 households (15/27; 55%) had non-index case individuals (people or pets) that were positive for ESBL-/ACBL-producing *Enterobacteriaceae*, with 11 (11/27; 41%) of these being households positive for ESBL- and/or plasmid-mediated ACBL-producing E. coli (Fig. 1). These 11 households formed the basis for the remainder of this study, and the whole genomes of all the ESBL- and/or ACBL-producing *Enterobacteriaceae* isolates from the individuals in these 11 households were sequenced (Table 1; Table S2).

**TABLE 1:** Summary of the *Enterobacteriaceae* isolates from 11 households

Household	Source of isolates	Bacterial species	Phylogroup(s)^*a*^
HH08	Urine index case	E. coli	D
	Other person (*n* = 6)	E. coli	B1 (1/6), D (5/6)
HH15	Urine index case	E. coli	D
	Other person (*n* = 6)	E. coli	D (6/6)
HH16	Urine index case	E. coli	D
	Fecal index case (*n* = 8)	E. coli	D
	Other person (*n* = 5)	E. coli	D
HH24	Urine index case	E. coli	B2
	Fecal index case (*n* = 7)	E. coli	B2
	Other person (*n* = 6)	E. coli	B2 (4/6), F (2/6)
HH26	Urine index case	E. coli	A
	Fecal index case (*n* = 3)	E. coli	B2
	Pet dog (*n* = 8)	E. coli, *Citrobacter* sp.	B2 (7/8)
HH39	Urine index case	E. coli	B2
	Fecal index case (*n* = 4)	E. coli	B2
	Other person (*n* = 4)	E. coli	B2
HH40	Urine index case	E. coli	B2
	Fecal index case (*n* = 6)	E. coli	B2
	Other person 1 (*n* = 6)	E. coli	B2
	Other person 2 (*n* = 7)	E. coli	B2
	Other person 3 (*n* = 6)	E. coli	B2
HH48	Urine index case	E. coli	D
	Fecal index case (*n* = 4)	E. coli	D
	Pet dog (*n* = 4)	E. coli	D (2/4), B2 (1/4), NT (1/4)
HH64	Urine index case	E. coli	B1
	Pet dog (*n* = 4)	E. coli	B1 (3/4), D (1/4)
	Pet cat (*n* = 5)	*Enterobacter* spp.	
HH65	Urine index case	E. col	D
	Fecal index case (*n* = 7)	E. coli	D
	Pet dog (*n* = 5)	E. coli	A (4/5), B1 (1/5)
HH86	Urine index case	E. coli	B2
	Fecal index case (*n* = 1)	E. coli	B2
	Pet dog (*n* = 6)	E. coli	B2

a Values in parentheses represent the number of isolates of the indicated phylogroup/ total number of isolates from the individual.

### Genetic diversity of ESBL-producing *Enterobacteriaceae*.

**FIG 2:**
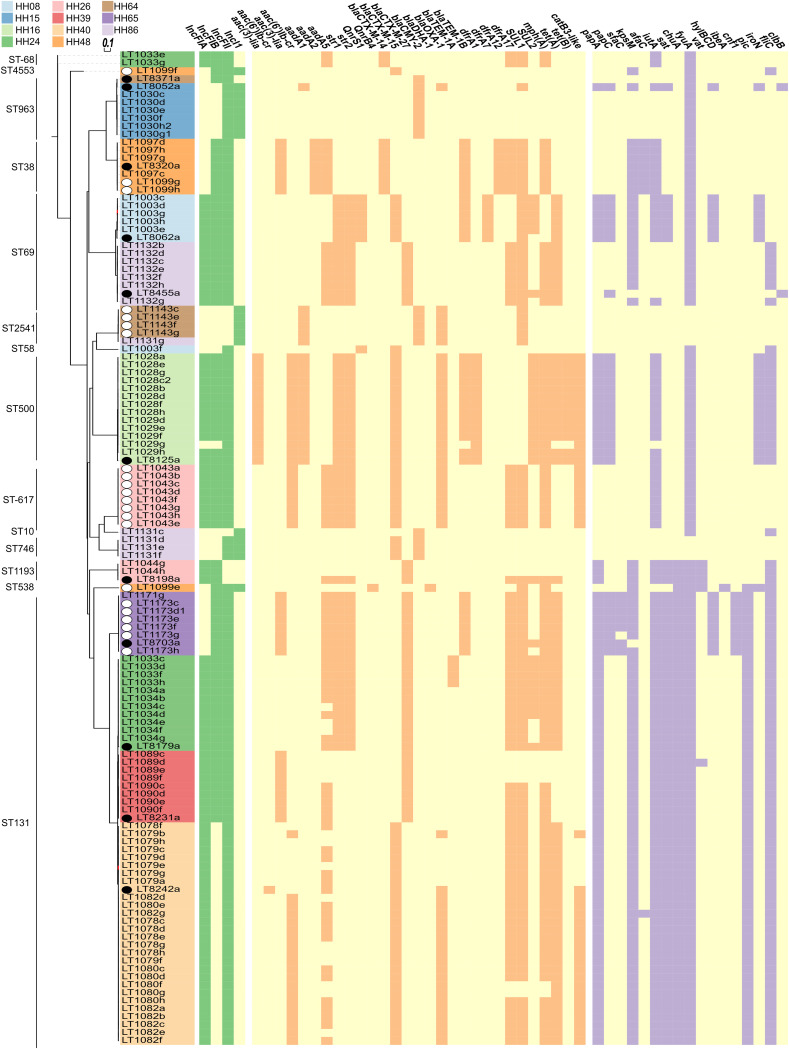
Whole-genome MLST of 125 E. coli isolates from 11 households. A total of 3,022 loci were used to construct a distance matrix using the Fast-GeP (v.1.0) genome profiler and the reference genome LT1099f. A black circle to the left of the tip label indicates that the isolate was from the index case urine sample, and a white circle indicates that the isolate originated from a dog fecal sample. The colored strip in the tip label (isolate identifier) denotes the household. The presence or absence of a plasmid, resistance, and the selected virulence genes was found using the PlasmidFinder (green/yellow heat map), ResFinder (orange/yellow heat map), and VirulenceFinder (purple/yellow heat map) databases, respectively.

In order to obtain the predominant ESBL-producing *Enterobacteriaceae* strain(s) associated with the households sampled, we cultured up to eight colonies per fecal sample as well as one colony from the index case urine sample. E. coli was the predominant species identified (125/131; 95%), with a *Citrobacter* species (1/131; 0.8%) and *Enterobacter* species (5/131; 4%) also being isolated. E. coli was the only bacterial species isolated that was shared between the index case and other members of the household (both people and pets). Short-read sequencing was carried out on all of the 125 E. coli strains from the 11 households that had an ESBL- or ACBL-producing phenotype (Table S2), and the draft whole-genome sequence assemblies were used to determine the phylogroup and sequence type of each isolate (Table 1), as well as to identify virulence factors, antibiotic resistance determinants, and plasmid types (Fig. 2). The sequence types (determined by use of the Nullarbor bioinformatics pipeline) ST131, ST69, and ST963 were found in more than one household; ST131 was found in four households and accounted for 47/125 (46%) of the isolates described here.

We next investigated the genetic relatedness of these strains by performing whole-genome multilocus sequence typing (wgMLST) for all 125 E. coli isolates, using 3,022 loci. The household-level patterns of virulence, antimicrobial resistance, and plasmid genes are also illustrated (Fig. 2). Isolates from five of the households represented distinct monophyletic clades. Analysis of the presence/absence of 25 virulence-associated genes (Fig. 2; Table S3) showed that the within-household clonal fecal and index urine strains had a similar virulence gene profile. Both the index urine and fecal isolates displayed some of the genes typically associated with extraintestinal pathogenic E. coli strains, such as *papA* (81/125; 65%), *papC* (30/125; 24%), *sfaC* (7/125; 6%), *afaC* (8/125; 6%), *kpsM* (82/125; 66%), and *iutA* (98/125; 78%) (31), as well as uropathogenic E. coli strain-associated genes such, as *sat* (67/125; 54%), *vat* (5/125; 4%), *fyuA* (117/125; 94%), and *chuA* (61/125; 49%) (31).

### Distribution of antimicrobial-resistant genotypes and phenotypes.

All the E. coli isolates were resistant to cefpodoxime, in agreement with their ESBL- or ACBL-producing phenotype (Table S4), and they all carried an ESBL gene (*bla*_CTX-M_) or an ACBL gene (*bla*_CMY_ or *bla*_DHA-1_) (Table 2 and Table S5). In some individuals and households, the same beta-lactamase gene variant was found across multiple sequence types. For example, the *bla*_CMY-2_ gene was associated with both ST10 and ST746 in one individual from household 65 (HH65), and in HH64, this gene was associated with the index urine ST963 isolate and a dog ST2541 isolate. An MDR phenotype was observed in 87 isolates (87/125; 70%), and an MDR genotype, where genes associated with resistance to three or more classes of antibiotics were detected (including genes that we did not test for phenotypically), was observed in 106 isolates (106/125; 85%) (Table 2). No colistin or carbapenem resistance genes were found.

**TABLE 2:** Resistance phenotype and genotype of E. coli isolates^*a*^

Household	Isolate(s)	Sequence type	Beta-lactamase phenotype	ESBL gene(s)	ACBL gene(s)	Other resistance phenotype	Other resistance gene(s)
HH08	LT8062a, LT1003c, LT1003d, LT1003e, LT1003g, LT1003h	ST69	CTX, AMP, LEX, CPD, CRO	*bla*_CTX-M-15_	None	TMP	*dfrA7*, *strA*, *strB*, *sul1*, *sul2*, *tetA*, *qnrS1*
	LT1003f	ST58	CTX, AMP, LEX, CPD, CRO	*bla*_CTX-M-15_	None	NIT	*qnrS1*
HH15	LT8052a	ST963	CTX, CAZ, AMP, AMC, LEX, CPD, CRO, FOX	None	*bla*_CMY-2_	NIT	*aadA1*-like, *sul1*
	LT1030c, LT1030d, LT1030e, LT1030f, LT1030g1, LT1030h2	ST963	CTX, CAZ, AMP, AMC, LEX, CPD, CRO, FOX	None	*bla*_CMY-2_	None	None
HH16	LT8125a, LT1028a, LT1028b, LT1028c2, LT1028d, LT1028e, LT1028f, LT1028g, LT1028h, LT1029d, LT1029e, LT1029f, LT1029g, LT1029h	ST500	CTX, CAZ, AMP, AMC, LEX, CPD, CRO, FOX, MEC	*bla*_CTX-M-15_	None	TMP, NIT, NOR, GEN	*aac(3)-IIa*-like, *aac(6′)Ib-cr*, *aadA1*, *catB3*-like, *dfrA1*, *mph*(A)-like, *strA*-like, *strB*-like, *sul2*-like, *tetA*, *tetB* (13/14)
HH24	LT8179a, LT1034g	ST131	CTX, AMP, LEX, CPD, CRO, MEC	*bla*_CTX-M-27_	None	TMP, NOR	*aadA5*, *dfrA17*, *mph*(A), *sul1*, *strA*, *strB*, *sul2*, *tetA*
	LT1033c, LT1033d, LT1034a, LT1034b, LT1034c, LT1034d, LT1034e, LT1034f	ST131	CTX, AMP, LEX, CPD, CRO	*bla*_CTX-M-27_	None	TMP, NOR	*aadA5*, *dfrA17*, *mph*(A), *sul1*, *strA*, *strB*, *sul2*, *tetA*
	LT1033h	ST131	CTX, AMP, LEX, CPD, CRO, MEC	*bla*_CTX-M-27_	None	TMP, NOR	*aadA5*, *dfrA17*, *mph*(A), *sul1*, *strA*, *strB*, *sul2*, *tetA*
	LT1033f	ST131	CTX, LEX, CPD, CRO, MEC	*bla*_CTX-M-27_	None	TMP, NOR	*aadA5*, *dfrA17*, *mph*(A), *sul1*, *strA*, *strB*, *sul2*, *tetA*
	LT1033e, LT1033g	ST648	CTX, AMP, LEX, CPD, CRO	*bla*_CTX-M-14_	None	TMP, NOR	*aadA5*, *dfrA17*, *mph*(A), *sul1*
HH26	LT1043a, LT1043b, LT1043d, LT1043e, LT1043f, LT1043g, LT1043h	ST617	CTX, CAZ, AMP, AMC, LEX, CPD, CRO	*bla*_CTX-M-15_	None	TMP, NIT, NOR	*aadA5*, *dfrA17*, *mph*(A), *strA*, *strB*-like, *sul1*, *sul2*, *aac(6′)Ib-cr*, *catB3*-like
	LT8198a	ST1193	CTX, AMP, LEX, CPD, CRO	*bla*_CTX-M-27_	None	TMP, NOR	*aadA5*, *dfrA17*, *mph*(A), *strA*, *strB*-like, *sul1*, *sul2*, *tetA*
	LT1043c	ST617	CTX, CAZ, AMC, LEX, CPD, CRO	*bla*_CTX-M-15_	None	TMP, NIT, NOR	*aadA5*, *dfrA17*, *mph*(A), *strA*, *strB*-like, *sul1*, *sul2*, *aac(6′)Ib-cr*, *catB3*-like
	LT1044g	ST1193	CTX, AMP, LEX, CPD, CRO	*bla*_CTX-M-27_	None	NOR	None
	LT1044h	ST1193	CTX, LEX, CPD, CRO	*bla*_CTX-M-27_	None	NOR	None
HH39	LT8231a, LT1090c, LT1090d, LT1090e, LT1090f	ST131	CTX, AMP, LEX, CPD, CRO	*bla*_CTX-M-27_	None	TMP, NOR, GEN	*aac(3)-IId*-like, *dfrA17*, *mph*(A), *sul1*
	LT1089c, LT1089d, LT1089e, LT1089f	ST131	CTX, AMP, LEX, CPD, CRO	*bla*_CTX-M-27_	None	NOR, GEN	*aac(3)-IId*-like
HH40	LT8242a, LT1082a, LT1082b, LT1082d, LT1082e, LT1082f, LT1082g, LT1080c, LT1080d, LT1080e, LT1080h, LT1079f, LT1079b, LT1078g, LT1078h, LT1078c, LT1078d, LT1078e	ST131	CTX, AMP, LEX, CPD, CRO	*bla*_CTX-M-15_	None	TMP, NOR	*aac(6′)Ib-cr*, *aadA5*, *catB3*-like, *dfrA17*, *mph*(A), *sul1*, *tetA*
	LT1078f, LT1079a, LT1079c, LT1079d, LT1079e, LT1079g, LT1079h	ST131	CTX, AMP, LEX, CPD, CRO	*bla*_CTX-M-15_	None	TMP, NOR	*aadA5*, *dfrA17*, *mph*(A), *sul1*, *tetA*
	LT1080f, LT1080g	ST131	CTX, AMP, LEX, CPD, CRO	*bla*_CTX-M-15_	None	NOR	*aac(3)-IId*-like, *catB3*-like, *tetA*
	LT1079b, LT1082c	ST131	CTX, AMP, LEX, CPD	*bla*_CTX-M-15_	None	TMP, NOR	*aac(6′)Ib-cr*, *aadA5*, *catB3*-like, *dfrA17*, *mph*(A), *sul1*, *tetA*
	LT1080d	ST131	CTX, CAZ, AMP, LEX, CPD, CRO	*bla*_CTX-M-15_	None	TMP, NOR	*aac(6′)Ib-cr*, *aadA5*, *catB3*-like, *dfrA17*, *mph*(A), *sul1*, *tetA*
HH48	LT1097c, LT1097d, LT1097h, LT1099g, LT1099h	ST38	CTX, AMP, LEX, CPD, CRO	*bla*_CTX-M-14_	None	TMP, GEN	*aac(3)-IId*-like, *aadA2*, *aadA5*, *dfrA12*, *dfrA17*, *mph*(A), *sul1*
	LT8320a	ST38	CTX, AMP, AMC, LEX, CPD, CRO	*bla*_CTX-M-14_	None	TMP, GEN	*aac(3)-IId*-like, *aadA2*, *aadA5*, *dfrA12*, *dfrA17*, *mph*(A), *sul1*
	LT1099e	ST538	CTX, CAZ, AMP, AMC, LEX, CPD, CRO, FOX	None	*bla*_DHA-1_	None	*mph*(A), *qnrB4*, *sul1*
	LT1099f	ST4553	CTX, AMP, LEX, CPD, CRO	*bla*_CTX-M-15_	None	None	None
	LT1097g	ST38	CTX, AMP, LEX, CPD, CRO	*bla*_CTX-M-14_	None	NIT, GEN	*aac(3)-IId*-like, *aadA2*, *aadA5*, *dfrA12*, *dfrA17*, *mph*(A), *sul1*
HH64	LT8371a	ST963	CTX, CAZ, AMP, AMC, LEX, CPD, CRO, FOX	None	*bla*_CMY-2_	NIT	None
	LT1143c, LT1143e, LT1143f, LT1143g	ST2541	CTX, CAZ, AMP, AMC, LEX, CPD, CRO, FOX	None	*bla*_CMY-2_	None	*aadA1*-like, *sul1*
HH65	LT1131d, LT1131e, LT1131f	ST746	CTX, CAZ, AMP, AMC, LEX, CPD, CRO, FOX	*bla*_CTX-M-15_	*bla*_CMY-2_	None	None
	LT1131c	ST10	CTX, CAZ, AMP, AMC, LEX, CPD, CRO, FOX	None	*bla*_CMY-2_	None	None
	LT1131g	ST2541	CTX, CAZ, AMP, AMC, LEX, CPD, CRO, FOX	None	*bla*_CMY-2_	None	*aadA1*-like, *sul1*
	LT8455a, LT1132b	ST69	CTX, AMP, LEX, CPD, CRO	*bla*_CTX-M-27_	None	TMP, NIT	*aadA5*, *dfrA17*, *mph*(A), *strA*, *strB*-like, *sul1*, *sul2*, *tetA*
	LT1132c, LT1132d, LT1132e, LT1132f, LT1132g, LT1132h	ST69	CTX, AMP, LEX, CPD, CRO	*bla*_CTX-M-27_	None	TMP	*aadA5*, *dfrA17*, *mph*(A), *strA*, *strB*-like, *sul1*, *sul2*, *tetA*
HH86	LT8703a	ST131	CTX, AMP, AMC, LEX, CPD, CRO	*bla*_CTX-M-27_	None	TMP, GEN	*aac(3)-IId*-like, *aadA5*, *dfrA17*, *mph*(A), *strA*, *strB*, *sul1*, *sul2*, *tetA*
	LT1171g, LT1173c, LT1173d, LT1173e, LT1173f, LT1173g, LT1173h	ST131	CTX, AMP, LEX, CPD, CRO	*bla*_CTX-M-27_	None	TMP, GEN	*aac(3)-IId*-like, *aadA5*, *dfrA17*, *mph*(A), *strA*, *strB*, *sul1*, *sul2*, *tetA*

a CTX, cefotaxime; CAZ, ceftazidime; AMP, ampicillin; AMC, amoxicillin/clavulanic acid; LEX, cephalexin; CPD, cefpodoxime; CRO, ceftriaxone; MEC, mecillinam; FOX, cefoxitin; TMP, trimethoprim; NIT, nitrofurantoin; NOR, norfloxacin; GEN, gentamicin.

### E. coli population within households.

**FIG 3:**
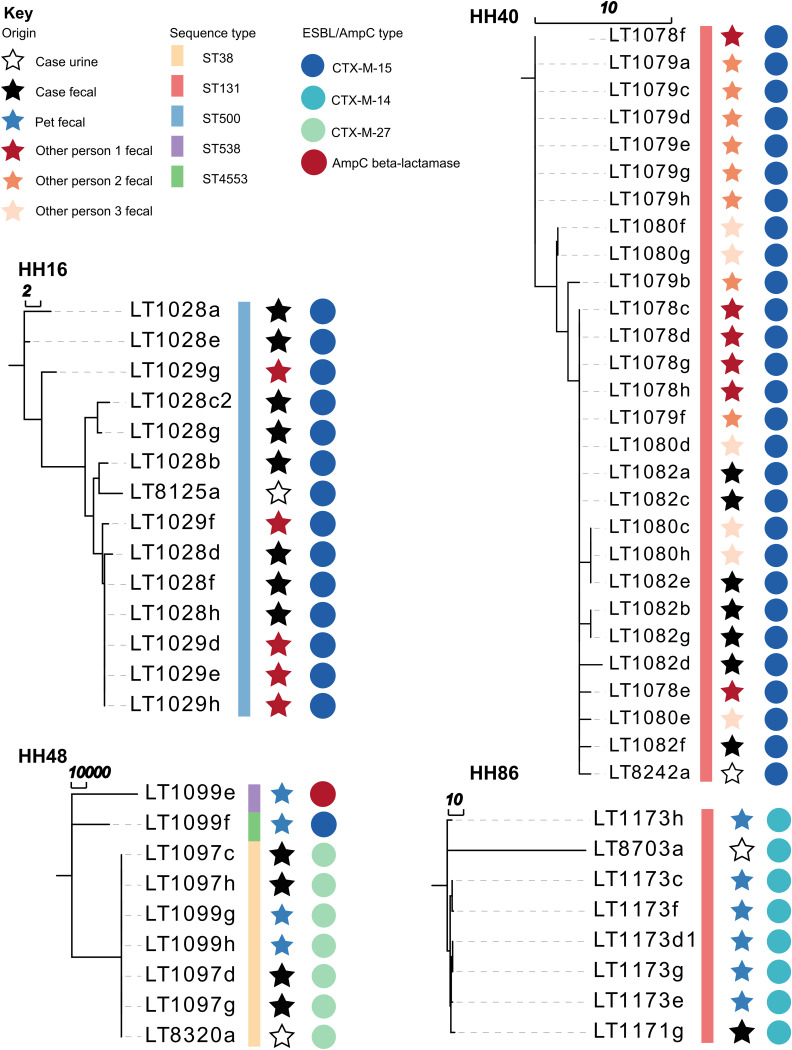
Neighbor-joining core SNP phylogenies, generated using the Snippy program (v.3.0), for ESBL-/ACBL-producing E. coli isolates from four households. The scale bar represents the branch length (in number of SNPs).

**FIG 4:**
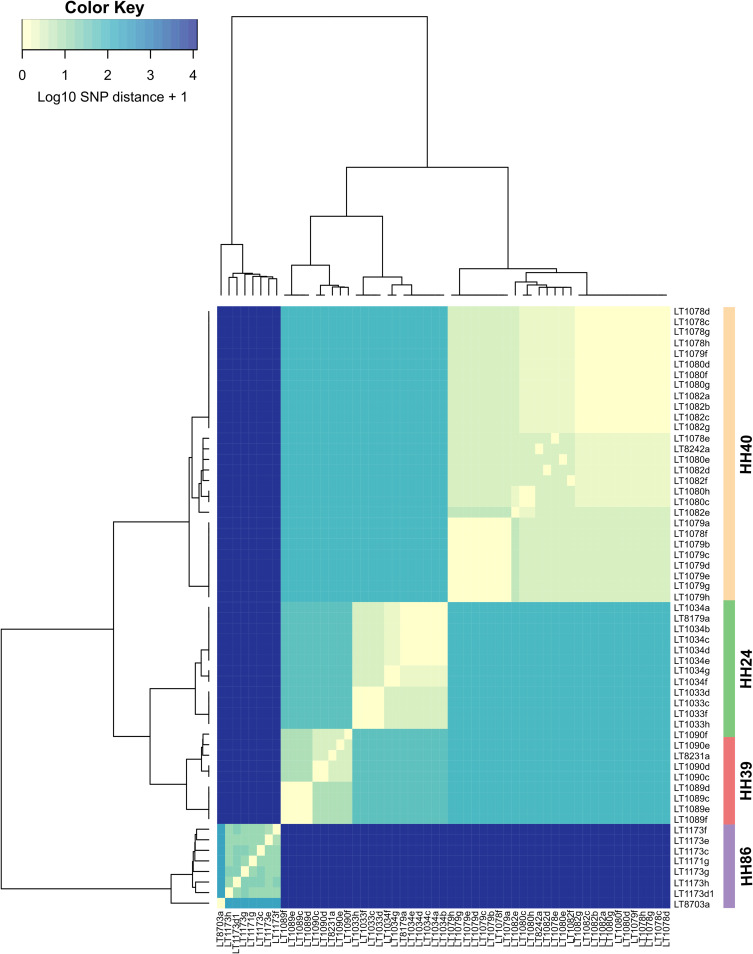
Dendrogram of the core SNP phylogeny of E. coli ST131 isolates from four households. The core SNP alignment was determined using 12,454 SNPs. Reference genome JJ1886 was used for core SNP phylogeny, using the Snippy program (v.3.0). The heat map represents the log + 1 of the number of SNPs between each pair of ST131 isolates.

To further explore the genetic relatedness of isolates within households, paired-end sequence reads from each E. coli isolate were compared to a draft assembled reference genome selected from that household (Table S6). These comparisons were made using core single nucleotide polymorphisms (SNPs), and the resultant trees for individual households are presented in Fig. 3 and Fig. S1. This demonstrated that in seven of the households (HH08, HH16, HH24, HH39, HH40, HH48, and HH86), clonal strains (strains with a difference of <10 SNPs) were cultured from more than one household member and from a person and a pet in two households (Fig. 3, HH48 and HH86). We also made comparisons using a SNP analysis of the E. coli ST131 isolates across four households (Fig. 4), as well as ST69 and ST963 isolates across two households (Table S7 and S8). These results demonstrated that clonal ST131, ST69, and ST963 isolates were cultured from more than one person or pet within a household but not across these households.

## DISCUSSION

### Fecal carriage of ESBL-producing *Enterobacteriaceae*.

This study is an important first step in examining the role of household contacts and pets in the transmission of community-acquired ESBL-/ACBL-producing E. coli strains associated with infections. The proportion of household members (9/18; 50% of people) that carried ESBL- or ACBL-producing *Enterobacteriaceae* was higher than that in other case-index carriage studies, which has been reported to range from 8.0% to 36.9% (32). Similarly, the proportion of companion animals that carried ESBL- or ACBL-producing *Enterobacteriaceae* (9/36 animals; 25%) was higher than that reported in previous studies of healthy companion animals, which ranged from 0 to 20.5% (33–35). In New Zealand, there are limited data on the prevalence of ESBL- and ACBL-producing *Enterobacteriaceae* from both healthy companion animals and clinical samples. The prevalence of ESBL-producing E. coli from healthy cats and dogs was reported to be 6.5% (30).

Intestinal carriage of MDR *Enterobacteriaceae* has been described to be a significant risk for subsequent infection with MDR bacteria (36, 37), and it is very likely that the proportion of carriers will continue to rise in the coming years (38). Benchmarking the prevalence of ESBL-producing bacteria in the community could be a useful first step for ongoing surveillance. This was not assessed in the current study, although it should be noted that 25% (3/12) of household human contacts carried an ESBL-producing E. coli strain unrelated to the index UTI in this study.

### Epidemiology of observed sequenced types.

The predominant sequence type of strains isolated from both urine and human fecal samples was ST131. It has been well established that this global lineage is the predominant cause of both UTIs and blood infections; additionally, it is commonly found in other human-derived samples, such as feces and sewage (39–41). Other sequence types found across multiple households were ST69 and ST963. ST69 has frequently been associated with UTIs (35, 36), whereas ST963 is rare in humans but has been detected in wild birds and companion animals (albeit in low numbers) (30, 42, 43). In the two households where ESBL-producing E. coli strains were shared by humans and their pet dogs, these strains belonged to ST131 as well as ST38, which, among ESBL-producing strains, is another lineage frequently associated with blood infections in humans (39). To our knowledge, most of the other STs found only in dogs in our study (ST1193, ST4553, ST746, ST2541, ST10) are rare, with the exception of ST10, which has frequently been found in both animals and humans (39, 44).

### Household transmission.

The sharing of ESBL- or ACBL-producing E. coli occurred between multiple household members and/or pets in 7/11 (64%) of the investigated households. However, this proportion varied according to whether the household contacts sampled were people or pets. In 50% (5/10) of the households with multiple people, clonal strains (strains with a difference of <10 SNPs) of ESBL-producing E. coli were cultured from more than one person in the household, whereas in 22% (2/9) of the households where pets were sampled, clonal strains were cultured from a person in the household and the pet. The results of this study are comparable to those of other studies, where the carriage of clonally related ESBL- or ACBL-producing *Enterobacteriaceae* has been found in a similar proportion of family members (27, 32, 45).

Pets have been described in previous studies as carrying the same E. coli clonal type as that carried by humans in the same household and in some cases being affected by clinical UTIs (27). The dogs that were positive for fecal ESBL-producing E. coli isolates in our study may have picked up these bacteria from raw meat, other animals, the environment, or their owners. Transmission may have occurred between dogs and their owners that shared the same strain; however, inference of the directionality of AMR transmission dynamics requires long-term sequential sampling. Another explanation for the sharing of strains is the acquisition of these bacteria from the same source by both the owner and the owner’s pet. However, this seems unlikely, given that an earlier New Zealand study did not find ESBL-producing E. coli in meat, and the survival of these bacteria on surfaces is limited (46, 47).

The results presented here show that pets are at least transient carriers of ESBL- and ACBL-producing *Enterobacteriaceae*. It is of note that none of the animals positive for the same strain of E. coli as the humans in the household were cats. In agreement with the findings of other studies (35), significantly more dogs than cats carried ESBL-/plasmid-mediated ACBL-producing E. coli strains. Cats and dogs behave differently with regard to both the type of contact with owners (less hand and face licking by cats) and food (for instance, cats are less likely to eat the same food as humans, including pieces of food handed over by owners during a meal). Although eating raw meat is a risk factor for ESBL-producing *Enterobacteriaceae* carriage in both dogs and cats (29, 35, 48), in our study, significantly more dogs than cats consumed raw meat as part of their diet.

Close contact within the home between partners, between parents and children, and between pets and their owners may be a factor in the transmission of the MDR bacteria isolated in this study. In New Zealand, clinical infections with MDR E. coli occur in pets but occur at rates that are unknown and that are assumed to be low (8, 49). Interestingly, isolates of E. coli collected from dog fecal samples in two different households had unique fecal AMR E. coli strains, all of which were of an ST different from that of the clinical case. While these STs are not commonly associated with community-acquired infection in New Zealand, the enzyme-coding genes (*bla*_CTX-M-15_, *bla*_CTX-M-14_) were those also found predominantly in human infections in New Zealand (7, 50).

### Horizontal gene transfer.

Analysis of the genome sequences presented here suggests that horizontal gene transfer (via plasmids) between bacteria may have occurred in some individuals. Discrete plasmids were not assembled through the bioinformatics pipeline; however, large numbers of plasmid-associated genes (and related plasmid types) were identified and reported. Although plasmid-mediated gene transfer was not explored experimentally within this study, there is the potential for this to have occurred with the ACBL gene *bla*_CMY-2_, which was associated with isolates of two different sequence types in HH64. This highlights the value of collecting and sequencing multiple isolates from fecal samples. Long-read sequencing of plasmids would be required to confirm and compare the plasmids isolated from different bacteria to make any conclusions regarding horizontal gene transfer. Evidence of the transmissibility of plasmids from cultured bacteria to a donor organism (via conjugation) would also be required for any assertions around the intraperson (and, therefore, interperson) transmission of resistance genetics to be made.

### Study limitations.

A limitation to the study is that transmission within households cannot be examined by a cross-sectional methodology. Consequently, this is often assessed through mathematical modeling, in addition to observational studies (42). Within a longitudinal study of the postacquisition carriage of ESBL-producing *Enterobacteriaceae*, results were used for a transmission model that calculated a 12% probability of household transmission of ESBL-producing *Enterobacteriaceae* from a positive person to a negative person in the home (51). The results presented in this study identify that some sharing of ESBL- and ACBL-producing bacteria within households is likely, even though transmission dynamics cannot be inferred from this cross-sectional study.

### Conclusions.

The results of this study demonstrate the sharing of the same ESBL-producing E. coli strains between household members. This suggests that the transmission of ESBL-producing E. coli occurred through contact between people (or people and pets) after an introduction event or through exposure to the same source of AMR bacterial isolate. Although our study suggests that companion animals (specifically, dogs) are carriers of ESBL- and/or ACBL-producing E. coli, their importance is hard to assess, and they are likely to be less important vectors than other people living in the home. From a public health perspective, the findings from our study reinforce current guidelines on hygiene practices within households and the importance of considering the entire household in control measures to reduce the spread of AMR enteric pathogens.

## MATERIALS AND METHODS

### Ethics.

Human ethics for this study was granted by the New Zealand Health and Disability Ethics Committee (HDEC) under reference 15/CEN/47; this study was part of a larger prospective case-control study looking at risks for community-acquired UTIs. Animal ethics for sampling via rectal swab from cats was granted by the Massey University Animal Ethics Committee under reference 15/35.

### Participant selection.

Index cases were recruited based on culture and antimicrobial sensitivity testing results of urine samples collected from persons suspected to have a UTI and submitted to Labtests Auckland (Healthscope) between 28 September 2015 and 5 September 2017. All urine samples came from the Auckland and Northland regions of New Zealand. Additionally, all of these samples had the growth of E. coli in the presence of pyuria (≥10 × 10^6^ white blood cells/liter), and the persons were considered to have a community-acquired UTI.

Index cases were eligible if they were 16 years of age or older, had no previous detection of an ESBL- or ACBL-producing *Enterobacteriaceae* isolate, had no record of being hospitalized overnight in the previous 12 months, and did not reside in an elder care facility or rest home. Seventy-two index cases were invited to submit fecal samples from themselves, as well as from any other members of the household, including cats or dogs that lived with them in the home. Five declined to participate; for the remaining 67, information on age, gender/sex, as well as antimicrobial treatment and hospitalization/veterinary care for the previous 6 months was collected.

### Bacterial culture, identification, and susceptibility testing.

Bacterial isolates from index case urine samples were collected from Labtests Auckland. Fecal samples were collected by the participants, using a sterile fecal collection bottle. Pet fecal samples were collected either by direct sampling with a rectal swab (cats only, where litter trays were not used), using an agar transport swab (Copan Diagnostics, Brescia, Italy), or by the participant from the ground or litter box. Samples were processed by plating onto culture media, as follows: plain MacConkey agar (BD Difco, supplied by Fort Richard Laboratories, Auckland, New Zealand), MacConkey agar with 1-mg/liter cefotaxime sodium (Sigma-Aldrich, St. Louis, MO, USA), MacConkey agar with 1-mg/liter ceftazidime pentahydrate (Sigma-Aldrich), and chromogenic ESBL CHROMagar (CAC; Becton, Dickinson, Heidelberg, Germany). Rectal swabs taken from cats were enriched in buffered peptone water for approximately 16 h at 35°C before being plated onto the culture media. The agar plates were incubated overnight at 35°C, and two morphologically different single colonies (from each agar type) were selected for subculture onto Columbia horse blood agar (Fort Richard Laboratories). Up to eight colonies were chosen for subculture from each fecal sample. Identification of bacterial species from both urine and feces was done using matrix-assisted laser desorption ionization–time of flight (MALDI-TOF) mass spectrometry (for fecal strains, the mass spectrometer was from bioMérieux, Marcy-l’Etoile, France; for urine strains, the mass spectrometer was from Bruker, Billerica, MA, USA).

Isolates were tested for antimicrobial susceptibility and an ESBL-producing phenotype according to EUCAST guidelines, using a Kirby-Bauer disk diffusion assay (52, 53). Isolates were also tested for an AmpC-producing phenotype using a three-disk comparison assay (D69C AmpC disk test; Mast Group Ltd., Liverpool, UK). Susceptibility to an additional 14 antibiotics (see Table S9 in the supplemental material) was carried out using the Kirby-Bauer disk diffusion assay, using EUCAST clinical breakpoints (52, 54). “Moderate” susceptibilities were defined as those that fell between the susceptible and resistance diameters for the following antibiotics: ceftriaxone, gentamicin, amikacin, norfloxacin, and trimethoprim.

### Genome sequencing and bioinformatics.

*Enterobacteriaceae* isolates (*n* = 131) were selected for whole-genome sequencing from 11 households where multiple household members were positive for the carriage of at least one ESBL- or ACBL-producing *Enterobacteriaceae* isolate. Extraction of genomic DNA was conducted using a QIAamp DNA minikit (Qiagen, Hilden, Germany), and libraries were prepared using a Nextera XT DNA library preparation kit (Illumina Inc., San Diego, CA, USA). Sequencing was performed using Illumina MiSeq 2 × 250-bp paired-end reads (by the Massey Genome Service, Massey University, Palmerston North, New Zealand) and Illumina HiSeq 2 × 125-bp paired-end reads (by the University of Otago Genetic Analysis Service, Dunedin, New Zealand). Raw sequence reads were assessed for quality and processed (using the QCtool Solexa++, PhiX adapter removal [55]), prior to assembly and analysis using the Nullarbor bioinformatics pipeline in the “accurate” mode (56). Assembly of genomes in this pipeline was performed using the SPAdes assembler (v.3.0) (57), while single nucleotide polymorphism (SNP) analysis was executed by the use of the Snippy program (v.3.0) (58). All assembled genomes were checked for quality of assembly using the outputs from Nullarbor (v.1.25) and Quast (v.4.5) software (59). These assembled genomes were uploaded to the Center for Genomic Epidemiology pipeline (http://www.genomicepidemiology.org), from which the plasmid type (determined using the PlasmidFinder database [v.2.0]) (60), resistance genes (determined using the ResFinder database [v.3.1]) (61), and virulence genes (determined using the VirulenceFinder database [v.2.0, 23 April 2019]) (62) were identified. Virulence genes were also identified using the ABRicate program (v.0.8.13) with the VirulenceFinder database (23 April 2019) (62).

Initially, SNP analysis of the E. coli isolates included in this study was performed using the genome of an ESBL-producing E. coli strain (strain JJ1886) originating from a urinary tract infection as the reference genome (63). Individual SNP alignments were subsequently repeated for households with internal references. Whole-genome multilocus sequence typing (wgMLST) was executed using Fast-GeP (v.1.0) gene prediction and comparison software (64). A Nexus tree output was constructed using the SplitsTree program (v.4.14.8) and then transformed in a neighbor-joining Newick tree (65, 66) for upload to the EvolView web server for annotation and presentation (67).

### Data availability.

The sequence reads generated from this study have been deposited in the NCBI Sequence Read Archive under BioProject accession number PRJNA600954.

## Supplementary Material

Supplemental file 1
