# Supplementary material for: Carriage of Extended-Spectrum-Beta-Lactamase- and AmpC Beta-Lactamase-Producing Escherichia coli Strains from Humans and Pets in the Same Households
Source: Appl Environ Microbiol. 2020 Nov 24;86(24):e01613-20. doi: 10.1128/AEM.01613-20 (PMC7688229; doi:10.1128/AEM.01613-20)
Supplement: Supplemental file 1 [file AEM.01613-20-s0001.pdf]

Table S1. Description of 27 households that submitted fecal samples from household members

Household	Number of fecal samples taken from each household		Oral AMU in the 6 months prior to sampling					Number of fecal samples that were positive for ESBL or AmpC producing Enterobacteriaceae		Total ESBL- or AmpC-producing Enterobacteriaceae isolates‡
	Total people *	Total pets	Index case#			Non-index case people	Pets	From in-contact people	From pets	
			< 1 week	1 week – 3 months	3 – 6 months					
HH03	2	0	CIP_NOR	None	None	Yes	No	0	-	1[e]
HH08	2	2[a]	TMP	None	None	No	Yes (LT1005)	1	0	7
HH15	2	0	TMP	AMX, TMP	CHL	Yes	-	1	-	7
HH16	2	0	None	TMP, NIT, CEC, DOX, MAC	DOX, CEC	Unsure	-	1	-	14
HH21	1	1[a]	NIT	None	None	-	No	-	0	2[e]
HH22	1	1[a]	None	MAC	None	-	No	-	0	2[e]
HH24	2	2[a,b]	LEX	None	MAC	None	Yes (LT1032[b])	1	0	14
HH26	1	1[b]	TMP	TMP, CIP_NOR	CIP_NOR	-	Yes (LT1043)	-	1[b]	12
HH35	2	4[a,b]	None	None	TMP	Unsure	Yes (LT1083[a])	0	0	1[e]
HH36	1	1[a]	TMP, CIP_NOR	TMP	None	-	No	-	0	3
HH38	3	1[a]	TMP	None	None	Yes (LT1064)	Yes (LT1063)	0	0	1
HH39	2	1[a]	NIT	TMP	TMP	Yes	No	1	0	10
HH40	4	3[a,b]	None	TMP	FLU_PEN, MAC, DOX	No [d]	No	3	0	28
HH45	1	1[b]	CIP_NOR	None	None	-	No	-	0	1[e]
HH47	2	0	NIT	None	None	No	-	1†	-	6
HH48	2	2[b]	CIP_NOR	TMP	TMP, AUG	No	Yes (LT1098, LT1099)	0	1[b]	9

HH49	1	1[b]	CEC, NIT,	CEC, CIP_NOR, NIT, TMP, AMC, SXT	None	-	No	-	0	1[e]
HH53	1	2[a,b]	TMP	TMP	None	-	Yes (LT1115[b])	-	1[a†]	11
HH60	2	2a,b	CEC	CEC, FLU_PEN	None	No	Unsure	0	0	2[e]
HH61	1	2a	NA	NA	NA	-	Yes (LT1135)	-	0	3[e]
HH64	2	2[a,b]	NA	NA	NA	Yes	Yes (LT1143[b])	0	2[a†, b]	10
HH65	2	1[,b]	NA	NA	NA	NA	No	0	1[b]	13
HH68	1	1[b]	NA	NA	NA	-	Yes	-	0	1[e]
HH71	1	1[a]	NA	NA	NA	-	No	-	1[a†]	12
HH77	1	1[b]	NA	NA	NA	-	No	-	1[b†]	9
HH85	1	1[b]	NA	NA	NA		No	-	0	8[e]
HH86	2	2[b]	NA	NA	NA	No	Yes (LT1172)	0	1[b]	8

* Includes fecal sample from index case; #AMX, amoxicillin; AMC, amoxycillin/clavulanic acid; CEC, cefaclor; LEX, cephalexin; CHL, chloramphenicol; CIP_NOR, ciprofloxacin or norfloxacin; DOX, doxycycline; FLU_PEN, flucloxacillin or penicillin; MAC, macrolide; NIT, nitrofurantoin; TMP, trimethoprim; SXT, trimethoprim sulfamethoxazole; ‡Includes isolate from case index urine sample; † Not *E. coli*
[a] cat; [b] dog; [c] from one in-contact person only; [d] all in-contact people, [e] isolates from index case only (urine and/or faecal isolates)

Households which cultured ESBL or ACBL-*E. coli* and were included in further genomic analysis are highlighted in grey.

Table S2 Description of sequencing and assembly of 125 *E. coli* isolates

Isolate ID	Source	Source Type	Household	Collection date	Species	MLST	Genome size (bp)	GC	Read depth	Contigs	N50
LT8062a	Human, case	Urine	HH08	9/11/2015	Escherichia coli	ST69	5492481	51.5	48	243	285535
LT1003c	Human, in-contact	Feces	HH08	13/12/2015	Escherichia coli	ST69	5434577	50.7	94	553	164042
LT1003d	Human, in-contact	Feces	HH08	13/12/2015	Escherichia coli	ST69	5445852	50.6	135	575	158266
LT1003e	Human, in-contact	Feces	HH08	13/12/2015	Escherichia coli	ST69	5436162	49	73	627	121170
LT1003f	Human, in-contact	Feces	HH08	13/12/2015	Escherichia coli	ST58	4864009	50.7	82	248	119956
LT1003g	Human, in-contact	Feces	HH08	13/12/2015	Escherichia coli	ST69	5434782	50.4	107	540	150367
LT1003h	Human, in-contact	Feces	HH08	13/12/2015	Escherichia coli	ST69	5443523	50.5	109	590	158266
LT8052a	Human, case	Urine	HH15	28/10/2015	Escherichia coli	ST963	5150269	50.5	134	320	115994
LT1030c	Human, in-contact	Feces	HH15	12/02/2016	Escherichia coli	ST963	5174159	50.5	113	288	94030
LT1030d	Human, in-contact	Feces	HH15	12/02/2016	Escherichia coli	ST963	5182980	50.1	125	298	97413
LT1030e	Human, in-contact	Feces	HH15	12/02/2016	Escherichia coli	ST963	5172726	50.1	83	269	94501
LT1030f	Human, in-contact	Feces	HH15	12/02/2016	Escherichia coli	ST963	5176389	50.2	59	279	93422
LT1030g1	Human, in-contact	Feces	HH15	12/02/2016	Escherichia coli	ST963	5171168	50	71	267	93422
LT1030h2	Human, in-contact	Feces	HH15	12/02/2016	Escherichia coli	ST963	5168471	50	61	249	117931
LT8125a	Human, case	Urine	HH16	15/12/2015	Escherichia coli	ST500	5270376	50.5	56	216	163953
LT1028a	Human, case	Feces	HH16	12/02/2016	Escherichia coli	ST500	5221272	49.2	105	402	112357
LT1028b	Human, case	Feces	HH16	12/02/2016	Escherichia coli	ST500	5254279	49.5	89	361	121189
LT1028c2	Human, case	Feces	HH16	12/02/2016	Escherichia coli	ST500	5215390	49.6	88	367	104399
LT1028d	Human, case	Feces	HH16	12/02/2016	Escherichia coli	ST500	5252332	49.5	114	358	121189
LT1028e	Human, case	Feces	HH16	12/02/2016	Escherichia coli	ST500	5207435	49.4	96	304	120914
LT1028f	Human, case	Feces	HH16	12/02/2016	Escherichia coli	ST500	5247202	50	144	327	121134
LT1028g	Human, case	Feces	HH16	12/02/2016	Escherichia coli	ST500	5216141	50	111	326	112357
LT1028h	Human, case	Feces	HH16	12/02/2016	Escherichia coli	ST500	5247855	50	122	345	121134
LT1029d	Human, in-contact	Feces	HH16	12/02/2016	Escherichia coli	ST500	5248669	49.9	147	316	119604
LT1029e	Human, in-contact	Feces	HH16	12/02/2016	Escherichia coli	ST500	5264378	49.9	141	346	112400
LT1029f	Human, in-contact	Feces	HH16	12/02/2016	Escherichia coli	ST500	5245042	49.9	133	312	104398
LT1029g	Human, in-contact	Feces	HH16	12/02/2016	Escherichia coli	ST500	5156463	49.7	103	298	121024
LT1029h	Human, in-contact	Feces	HH16	12/02/2016	Escherichia coli	ST500	5245579	50.1	158	336	104399
LT8179a	Human, case	Urine	HH24	28/01/2016	Escherichia coli	ST131	5160657	51.1	81	178	179969
LT1033c	Human, in-contact	Feces	HH24	11/03/2016	Escherichia coli	ST131	5161406	50.1	103	254	160632

LT1033d	Human, in-contact	Feces	HH24	11/03/2016	Escherichia coli	ST131	5165516	50	85	269	159842
LT1033e	Human, in-contact	Feces	HH24	11/03/2016	Escherichia coli	ST648	5274284	49.8	104	266	154029
LT1033f	Human, in-contact	Feces	HH24	11/03/2016	Escherichia coli	ST131	5173677	50.1	113	281	160608
LT1033g	Human, in-contact	Feces	HH24	11/03/2016	Escherichia coli	ST648	5274564	49.6	100	236	154186
LT1033h	Human, in-contact	Feces	HH24	11/03/2016	Escherichia coli	ST131	5150923	50.2	121	246	160462
LT1034a	Human, case	Feces	HH24	11/03/2016	Escherichia coli	ST131	5104681	50.3	139	224	160490
LT1034b	Human, case	Feces	HH24	11/03/2016	Escherichia coli	ST131	5113673	50.3	110	250	160353
LT1034c	Human, case	Feces	HH24	11/03/2016	Escherichia coli	ST131	5118124	50.6	294	248	160736
LT1034d	Human, case	Feces	HH24	11/03/2016	Escherichia coli	ST131	5113867	50.5	172	251	160703
LT1034e	Human, case	Feces	HH24	11/03/2016	Escherichia coli	ST131	5104204	50.4	101	213	160814
LT1034f	Human, case	Feces	HH24	11/03/2016	Escherichia coli	ST131	5108057	50.5	141	228	159114
LT1034g	Human, case	Feces	HH24	11/03/2016	Escherichia coli	ST131	5107222	50.5	94	227	160606
LT8198a	Human, case	Urine	HH26	5/02/2016	Escherichia coli	ST1193	5092266	50.3	130	574	162923
LT1043a	Dog	Feces	HH26	8/03/2016	Escherichia coli	ST617	4897051	50.3	156	344	87883
LT1043b	Dog	Feces	HH26	8/03/2016	Escherichia coli	ST617	4882334	50.2	112	284	90674
LT1043c	Dog	Feces	HH26	8/03/2016	Escherichia coli	ST617	4888721	50.4	164	294	87883
LT1043d	Dog	Feces	HH26	8/03/2016	Escherichia coli	ST617	4889993	50.3	196	310	88609
LT1043e	Dog	Feces	HH26	8/03/2016	Escherichia coli	ST617	4887791	50.4	100	282	90674
LT1043f	Dog	Feces	HH26	8/03/2016	Escherichia coli	ST617	4886047	50.5	157	282	88609
LT1043g	Dog	Feces	HH26	8/03/2016	Escherichia coli	ST617	4887243	50.3	121	303	88130
LT1043h	Dog	Feces	HH26	8/03/2016	Escherichia coli	ST617	4886315	50.3	135	289	87669
LT1044g	Human, case	Feces	HH26	8/03/2016	Escherichia coli	ST1193	4985966	50.3	118	219	136965
LT1044h	Human, case	Feces	HH26	8/03/2016	Escherichia coli	ST1193	4978130	49.7	95	199	136965
LT8231a	Human, case	Feces	HH39	4/03/2016	Escherichia coli	ST131	5206381	50.3	99	444	156192
LT1089c	Human, in-contact	Feces	HH39	7/05/2016	Escherichia coli	ST131	5246570	50.4	116	346	154224
LT1089d	Human, in-contact	Feces	HH39	7/05/2016	Escherichia coli	ST131	5245849	50.6	110	373	156060
LT1089e	Human, in-contact	Feces	HH39	7/05/2016	Escherichia coli	ST131	5241731	50.7	112	348	154224
LT1089f	Human, in-contact	Feces	HH39	7/05/2016	Escherichia coli	ST131	5244117	50.4	109	344	154224
LT1090c	Human, case	Feces	HH39	7/05/2016	Escherichia coli	ST131	5195527	51.1	115	375	135260
LT1090d	Human, case	Feces	HH39	7/05/2016	Escherichia coli	ST131	5189400	50.6	117	372	154224
LT1090e	Human, case	Feces	HH39	7/05/2016	Escherichia coli	ST131	5184690	50.1	90	333	135260
LT1090f	Human, case	Feces	HH39	7/05/2016	Escherichia coli	ST131	5188047	50.8	113	305	173795
LT8242a	Human, case	Urine	HH40	20/03/2016	Escherichia coli	ST131	5215701	51	181	447	170471
LT1078c	Human,	Feces	HH40	6/05/2016	Escherichia coli	ST131	5167731	49.8	57	228	170471

	in-contact										
LT1078d	Human, in-contact	Feces	HH40	6/05/2016	Escherichia coli	ST131	5163690	50.6	112	191	178679
LT1078e	Human, in-contact	Feces	HH40	6/05/2016	Escherichia coli	ST131	5164730	50.3	137	189	178679
LT1078f	Human, in-contact 1	Feces	HH40	6/05/2016	Escherichia coli	ST131	5164284	50.5	129	199	178679
LT1078g	Human, in-contact	Feces	HH40	6/05/2016	Escherichia coli	ST131	5165121	50.1	90	210	178679
LT1078h	Human, in-contact	Feces	HH40	6/05/2016	Escherichia coli	ST131	5168065	50.4	121	211	178679
LT1079a	Human, in-contact 2	Feces	HH40	6/05/2016	Escherichia coli	ST131	5166581	50.4	102	219	178679
LT1079b	Human, in-contact 2	Feces	HH40	6/05/2016	Escherichia coli	ST131	5169484	50.5	123	206	178679
LT1079c	Human, in-contact 2	Feces	HH40	6/05/2016	Escherichia coli	ST131	5168641	50.4	256	244	170471
LT1079d	Human, in-contact 2	Feces	HH40	6/05/2016	Escherichia coli	ST131	5165281	50.4	136	216	178679
LT1079e	Human, in-contact 2	Feces	HH40	6/05/2016	Escherichia coli	ST131	5165605	50.4	142	205	178679
LT1079f	Human, in-contact 2	Feces	HH40	6/05/2016	Escherichia coli	ST131	5169293	50.5	136	202	178679
LT1079g	Human, in-contact 2	Feces	HH40	6/05/2016	Escherichia coli	ST131	5172039	50.6	161	241	178679
LT1079h	Human, in-contact 2	Feces	HH40	6/05/2016	Escherichia coli	ST131	5162297	50.2	90	204	178679
LT1080c	Human, in-contact 3	Feces	HH40	6/05/2016	Escherichia coli	ST131	5176850	50.4	140	243	178679
LT1080d	Human, in-contact 3	Feces	HH40	6/05/2016	Escherichia coli	ST131	5172358	50.4	133	220	180448
LT1080e	Human, in-contact 3	Feces	HH40	6/05/2016	Escherichia coli	ST131	5172620	50.3	212	233	178679
LT1080f	Human, in-contact 3	Feces	HH40	6/05/2016	Escherichia coli	ST131	5152282	50.4	132	197	178679
LT1080g	Human, in-contact 3	Feces	HH40	6/05/2016	Escherichia coli	ST131	5154204	50.3	58	204	178679
LT1080h	Human, in-contact 3	Feces	HH40	6/05/2016	Escherichia coli	ST131	5190026	50.8	175	312	181987
LT1082a	Human, case	Feces	HH40	6/05/2016	Escherichia coli	ST131	5173724	50.7	154	237	180448
LT1082b	Human, case	Feces	HH40	6/05/2016	Escherichia coli	ST131	5178377	50.7	180	253	180448
LT1082c	Human, case	Feces	HH40	6/05/2016	Escherichia coli	ST131	5170299	50.2	86	219	178679
LT1082d	Human, case	Feces	HH40	6/05/2016	Escherichia coli	ST131	5168602	50.5	124	211	180448
LT1082e	Human, case	Feces	HH40	6/05/2016	Escherichia coli	ST131	5167410	50.8	114	200	178679

LT1082f	Human, case	Feces	HH40	6/05/2016	Escherichia coli	ST131	5164692	50.3	107	181	170471
LT1082g	Human, case	Feces	HH40	6/05/2016	Escherichia coli	ST131	5172487	51.3	138	223	178679
LT8320a	Human, case	Urine	HH48	9/05/2016	Escherichia coli	ST38	5161032	51	173	381	123639
LT1097c	Human, case	Feces	HH48	26/06/2016	Escherichia coli	ST38	5158294	51	111	349	129502
LT1097d	Human, case	Feces	HH48	26/06/2016	Escherichia coli	ST38	5189465	51.2	207	516	127781
LT1097g	Human, case	Feces	HH48	26/06/2016	Escherichia coli	ST38	5151025	51.1	129	339	127890
LT1097h	Human, case	Feces	HH48	26/06/2016	Escherichia coli	ST38	5163741	51.1	199	371	129722
LT1099e	Dog	Feces	HH48	26/06/2016	Escherichia coli	ST538	5099672	50.3	116	189	218404
LT1099f	Dog	Feces	HH48	26/06/2016	Escherichia coli	ST4553	5285110	50	92	173	196514
LT1099g	Dog	Feces	HH48	26/06/2016	Escherichia coli	ST38	5151070	50.7	104	344	127952
LT1099h	Dog	Feces	HH48	26/06/2016	Escherichia coli	ST38	5147806	51.1	138	301	127778
LT8371a	Human, case	Urine	HH64	31/05/2016	Escherichia coli	ST963	5153473	50.2	120	338	92286
LT1143c	Dog	Feces	HH64	24/09/2016	Escherichia coli	ST2541	4969605	50.2	322	212	183218
LT1143e	Dog	Feces	HH64	24/09/2016	Escherichia coli	ST2541	4973592	51	90	230	192896
LT1143f	Dog	Feces	HH64	24/09/2016	Escherichia coli	ST2541	4969026	50.2	97	205	192896
LT1143g	Dog	Feces	HH64	24/09/2016	Escherichia coli	ST2541	4998195	50.1	103	354	175489
LT8455a	Human, case	Urine	HH65	25/07/2016	Escherichia coli	ST69	5120029	50.2	151	282	164000
LT1131c	Dog	Feces	HH65	24/09/2016	Escherichia coli	ST746	4914812	49.6	129	313	97240
LT1131d	Dog	Feces	HH65	24/09/2016	Escherichia coli	ST10	4811550	50.7	149	243	147407
LT1131e	Dog	Feces	HH65	24/09/2016	Escherichia coli	ST746	4902883	49.7	130	270	97240
LT1131f	Dog	Feces	HH65	24/09/2016	Escherichia coli	ST746	4900803	49.9	119	256	97240
LT1131g	Dog	Feces	HH65	24/09/2016	Escherichia coli	ST2541	5000520	49.4	112	325	175489
LT1132b	Human, case	Feces	HH65	24/09/2016	Escherichia coli	ST69	5122019	50.4	127	287	164000
LT1132c	Human, case	Feces	HH65	24/09/2016	Escherichia coli	ST69	5127711	50.3	121	318	164000
LT1132d	Human, case	Feces	HH65	24/09/2016	Escherichia coli	ST69	5124906	49.8	97	302	164000
LT1132e	Human, case	Feces	HH65	24/09/2016	Escherichia coli	ST69	5120564	50	122	271	164000
LT1132f	Human, case	Feces	HH65	24/09/2016	Escherichia coli	ST69	5125099	50.1	104	307	158267
LT1132g	Human, case	Feces	HH65	24/09/2016	Escherichia coli	ST69	5118820	50.8	134	288	164000
LT1132h	Human, case	Feces	HH65	24/09/2016	Escherichia coli	ST69	5127494	49.8	115	304	163970
LT1173f	Dog	Feces	HH086	14/01/2017	Escherichia coli	ST131	5165048	50.3	102	422	180447
LT1173g	Dog	Feces	HH086	14/01/2017	Escherichia coli	ST131	5141995	49.9	100	311	186438
LT1171g	Human, case	Feces	HH086	14/01/2017	Escherichia coli	ST131	5175922	50.7	92	449	180446
LT1173c	Dog	Feces	HH086	14/01/2017	Escherichia coli	ST131	5141055	50	100	301	190943

LT1173d1	Dog	Feces	HH086	14/01/2017	Escherichia coli	ST131	5148741	50.2	116	354	180448
LT1173e	Dog	Feces	HH086	14/01/2017	Escherichia coli	ST131	5138126	50.1	119	298	180447
LT1173h	Dog	Feces	HH086	14/01/2017	Escherichia coli	ST131	5144385	50.2	128	332	186560
LT8703a	Human, case	Urine	HH086	29/11/2016	Escherichia coli	ST131	5083072	50.6	131	443	178654

Table S3 Presence of virulence genes in 125 *E. coli* isolates from 11 households

Isolate	*papA*	*papH*	*papC*	*papEF*	*papG*	*sfaC*	*kpsM*	*afaC*	*iutA*	*sat*	*chuA*	*fyuA*	*vat*	*draD*	*hlyA*	*hlyBCD*	*ibeA*	*cnf1*	*pic*	*iroN*	*fliC*	*clbB*
LT1003c	+	+	+	+	+	ND	+	ND	+	+	ND	+	ND	ND	+	+	ND	ND	ND	+	ND	ND
LT1003d	+	+	+	+	+	ND	+	ND	+	+	ND	+	ND	ND	+	+	ND	ND	ND	+	ND	ND
LT1003e	+	+	+	+	+	ND	+	ND	+	+	ND	+	ND	ND	+	+	ND	ND	ND	+	ND	ND
LT1003f	ND	ND	ND	ND	ND	ND	ND	ND	ND	ND	ND	+	ND	ND	ND	ND	ND	ND	ND	ND	+	ND
LT1003g	+	+	+	+	+	ND	+	ND	+	+	ND	+	ND	ND	+	+	ND	ND	ND	+	ND	ND
LT1003h	+	+	+	+	+	ND	+	ND	+	+	ND	+	ND	ND	+	+	ND	ND	ND	+	ND	ND
LT1028a	+	+	+	+	+	ND	ND	ND	+	ND	ND	+	ND	ND	ND	ND	ND	ND	ND	+	+	ND
LT1028b	+	+	+	+	+	ND	ND	ND	+	ND	ND	+	ND	ND	ND	ND	ND	ND	ND	+	+	ND
LT1028c2	+	+	+	+	+	ND	ND	ND	+	ND	ND	+	ND	ND	ND	ND	ND	ND	ND	+	+	ND
LT1028d	+	+	+	+	+	ND	ND	ND	+	ND	ND	+	ND	ND	ND	ND	ND	ND	ND	+	+	ND
LT1028e	+	+	+	+	+	ND	ND	ND	+	ND	ND	+	ND	ND	ND	ND	ND	ND	ND	+	+	ND
LT1028f	+	+	+	+	+	ND	ND	ND	+	ND	ND	+	ND	ND	ND	ND	ND	ND	ND	+	+	ND
LT1028g	+	+	+	+	+	ND	ND	ND	+	ND	ND	+	ND	ND	ND	ND	ND	ND	ND	+	+	ND
LT1028h	+	+	+	+	+	ND	ND	ND	+	ND	ND	+	ND	ND	ND	ND	ND	ND	ND	+	+	ND
LT1029d	+	+	+	+	+	ND	ND	ND	+	ND	ND	+	ND	ND	ND	ND	ND	ND	ND	+	+	ND
LT1029e	+	+	+	+	+	ND	ND	ND	+	ND	ND	+	ND	ND	ND	ND	ND	ND	ND	+	+	ND
LT1029f	+	+	+	+	+	ND	ND	ND	+	ND	ND	+	ND	ND	ND	ND	ND	ND	ND	+	+	ND
LT1029g	+	+	+	+	+	ND	ND	ND	ND	ND	ND	+	ND	ND	ND	ND	ND	ND	ND	+	+	ND
LT1029h	+	+	+	+	+	ND	ND	ND	+	ND	ND	+	ND	ND	ND	ND	ND	ND	ND	+	+	ND
LT1030c	ND	ND	ND	ND	ND	ND	ND	ND	ND	ND	ND	+	ND	ND	ND	ND	ND	ND	ND	ND	ND	ND
LT1030d	ND	ND	ND	ND	ND	ND	ND	ND	ND	ND	ND	+	ND	ND	ND	ND	ND	ND	ND	ND	ND	ND
LT1030e	ND	ND	ND	ND	ND	ND	ND	ND	ND	ND	ND	+	ND	ND	ND	ND	ND	ND	ND	ND	ND	ND
LT1030f	ND	ND	ND	ND	ND	ND	ND	ND	ND	ND	ND	+	ND	ND	ND	ND	ND	ND	ND	ND	ND	ND
LT1030g+	ND	ND	ND	ND	ND	ND	ND	ND	ND	ND	ND	+	ND	ND	ND	ND	ND	ND	ND	ND	ND	ND
LT1030h2	ND	ND	ND	ND	ND	ND	ND	ND	ND	ND	ND	+	ND	ND	ND	ND	ND	ND	ND	ND	ND	ND

LT1033c	+	ND	ND	ND	ND	ND	+	ND	+	+	+	+	ND	ND	ND	ND	ND	ND	+	ND	+	ND
LT1033d	+	ND	ND	ND	ND	ND	+	ND	+	+	+	+	ND	ND	ND	ND	ND	ND	+	ND	+	ND
LT1033e	ND	ND	ND	ND	ND	ND	ND	ND	+	ND	ND	+	ND	ND	ND	ND	ND	ND	ND	ND	ND	ND
LT1033f	+	ND	ND	ND	ND	ND	+	ND	+	+	+	+	ND	ND	ND	ND	ND	ND	+	ND	+	ND
LT1033g	ND	ND	ND	ND	ND	ND	ND	ND	+	ND	ND	+	ND	ND	ND	ND	ND	ND	ND	ND	ND	ND
LT1033h	+	ND	ND	ND	ND	ND	+	ND	+	+	+	+	ND	ND	ND	ND	ND	ND	+	ND	+	ND
LT1034a	+	ND	ND	ND	ND	ND	+	ND	+	+	+	+	ND	ND	ND	ND	ND	ND	+	ND	+	ND
LT1034b	+	ND	ND	ND	ND	ND	+	ND	+	+	+	+	ND	ND	ND	ND	ND	ND	+	ND	+	ND
LT1034c	+	ND	ND	ND	ND	ND	+	ND	+	+	+	+	ND	ND	ND	ND	ND	ND	+	ND	+	ND
LT1034d	+	ND	ND	ND	ND	ND	+	ND	+	+	+	+	ND	ND	ND	ND	ND	ND	+	ND	+	ND
LT1034e	+	ND	ND	ND	ND	ND	+	ND	+	+	+	+	ND	ND	ND	ND	ND	ND	+	ND	+	ND
LT1034f	+	ND	ND	ND	ND	ND	+	ND	+	+	+	+	ND	ND	ND	ND	ND	ND	+	ND	+	ND
LT1034g	+	ND	ND	ND	ND	ND	+	ND	+	+	+	+	ND	ND	ND	ND	ND	ND	+	ND	+	ND
LT1043a	ND	ND	ND	ND	ND	ND	ND	ND	+	ND	ND	+	ND	ND	ND	ND	ND	ND	ND	ND	ND	ND
LT1043b	ND	ND	ND	ND	ND	ND	ND	ND	+	ND	ND	+	ND	ND	ND	ND	ND	ND	ND	ND	ND	ND
LT1043c	ND	ND	ND	ND	ND	ND	ND	ND	+	ND	ND	+	ND	ND	ND	ND	ND	ND	ND	ND	ND	ND
LT1043d	ND	ND	ND	ND	ND	ND	ND	ND	+	ND	ND	+	ND	ND	ND	ND	ND	ND	ND	ND	ND	ND
LT1043e	ND	ND	ND	ND	ND	ND	ND	ND	+	ND	ND	+	ND	ND	ND	ND	ND	ND	ND	ND	ND	ND
LT1043f	ND	ND	ND	ND	ND	ND	ND	ND	+	ND	ND	+	ND	ND	ND	ND	ND	ND	ND	ND	ND	ND
LT1043g	ND	ND	ND	ND	ND	ND	ND	ND	+	ND	ND	+	ND	ND	ND	ND	ND	ND	ND	ND	ND	ND
LT1043h	ND	ND	ND	ND	ND	ND	ND	ND	+	ND	ND	+	ND	ND	ND	ND	ND	ND	ND	ND	ND	ND
LT1044g	+	ND	ND	ND	ND	ND	+	ND	+	+	+	+	+	ND	ND	ND	ND	ND	ND	ND	+	ND
LT1044h	+	ND	ND	ND	ND	ND	+	ND	+	+	+	+	+	ND	ND	ND	ND	ND	ND	ND	+	ND
LT1078c	+	ND	ND	ND	ND	ND	+	ND	+	+	+	+	ND	ND	ND	ND	ND	ND	+	ND	+	ND
LT1078d	+	ND	ND	ND	ND	ND	+	ND	+	+	+	+	ND	ND	ND	ND	ND	ND	+	ND	+	ND
LT1078e	+	ND	ND	ND	ND	ND	+	ND	+	+	+	+	ND	ND	ND	ND	ND	ND	+	ND	+	ND
LT1078f	+	ND	ND	ND	ND	ND	+	ND	+	+	+	+	ND	ND	ND	ND	ND	ND	+	ND	+	ND
LT1078g	+	ND	ND	ND	ND	ND	+	ND	+	+	+	+	ND	ND	ND	ND	ND	ND	+	ND	+	ND
LT1078h	+	ND	ND	ND	ND	ND	+	ND	+	+	+	+	ND	ND	ND	ND	ND	ND	+	ND	+	ND

LT1079a	+	ND	ND	ND	ND	ND	+	ND	+	+	+	+	ND	ND	ND	ND	ND	ND	+	ND	+	ND
LT1079b	+	ND	ND	ND	ND	ND	+	ND	+	+	+	+	ND	ND	ND	ND	ND	ND	+	ND	+	ND
LT1079c	+	ND	ND	ND	ND	ND	+	ND	+	+	+	+	ND	ND	ND	ND	ND	ND	+	ND	+	ND
LT1079d	+	ND	ND	ND	ND	ND	+	ND	+	+	+	+	ND	ND	ND	ND	ND	ND	+	ND	+	ND
LT1079e	+	ND	ND	ND	ND	ND	+	ND	+	+	+	+	ND	ND	ND	ND	ND	ND	+	ND	+	ND
LT1079f	+	ND	ND	ND	ND	ND	+	ND	+	+	+	+	ND	ND	ND	ND	ND	ND	+	ND	+	ND
LT1079g	+	ND	ND	ND	ND	ND	+	ND	+	+	+	+	ND	ND	ND	ND	ND	ND	+	ND	+	ND
LT1079h	+	ND	ND	ND	ND	ND	+	ND	+	+	+	+	ND	ND	ND	ND	ND	ND	+	ND	+	ND
LT1080c	+	ND	ND	ND	ND	ND	+	ND	+	+	+	+	ND	ND	ND	ND	ND	ND	+	ND	+	ND
LT1080d	+	ND	ND	ND	ND	ND	+	ND	+	+	+	+	ND	ND	ND	ND	ND	ND	+	ND	+	ND
LT1080e	+	ND	ND	ND	ND	ND	+	ND	+	+	+	+	ND	ND	ND	ND	ND	ND	+	ND	+	ND
LT1080f	+	ND	ND	ND	ND	ND	+	ND	+	+	+	+	ND	ND	ND	ND	ND	ND	+	ND	+	ND
LT1080g	+	ND	ND	ND	ND	ND	+	ND	+	+	+	+	ND	ND	ND	ND	ND	ND	+	ND	+	ND
LT1080h	+	ND	ND	ND	ND	ND	+	ND	+	+	+	+	ND	ND	ND	ND	ND	ND	+	ND	+	ND
LT1082a	+	ND	ND	ND	ND	ND	+	ND	+	+	+	+	ND	ND	ND	ND	ND	ND	+	ND	+	ND
LT1082b	+	ND	ND	ND	ND	ND	+	ND	+	+	+	+	ND	ND	ND	ND	ND	ND	+	ND	+	ND
LT1082c	+	ND	ND	ND	ND	ND	+	ND	+	+	+	+	ND	ND	ND	ND	ND	ND	+	ND	+	ND
LT1082d	+	ND	ND	ND	ND	ND	+	ND	+	+	+	+	ND	ND	ND	ND	ND	ND	+	ND	+	ND
LT1082e	+	ND	ND	ND	ND	ND	+	ND	+	+	+	+	ND	ND	ND	ND	ND	ND	+	ND	+	ND
LT1082f	+	ND	ND	ND	ND	ND	+	ND	+	+	+	+	ND	ND	ND	ND	ND	ND	+	ND	+	ND
LT1082g	+	ND	ND	ND	ND	ND	+	+	+	+	+	+	ND	+	ND	ND	ND	ND	+	ND	+	ND
LT1089c	+	ND	ND	ND	ND	ND	+	ND	+	+	+	+	ND	ND	ND	ND	ND	ND	+	ND	+	ND
LT1089d	+	ND	ND	ND	ND	ND	+	ND	+	+	+	+	+	ND	ND	ND	ND	ND	+	ND	+	ND
LT1089e	+	ND	ND	ND	ND	ND	+	ND	+	+	+	+	ND	ND	ND	ND	ND	ND	+	ND	+	ND
LT1089f	+	ND	ND	ND	ND	ND	+	ND	+	+	+	+	ND	ND	ND	ND	ND	ND	+	ND	+	ND
LT1090c	+	ND	ND	ND	ND	ND	+	ND	+	+	+	+	ND	ND	ND	ND	ND	ND	+	ND	+	ND
LT1090d	+	ND	ND	ND	ND	ND	+	ND	+	+	+	+	ND	ND	ND	ND	ND	ND	+	ND	+	ND
LT1090e	+	ND	ND	ND	ND	ND	+	ND	+	+	+	+	ND	ND	ND	ND	ND	ND	+	ND	+	ND
LT1090f	+	ND	ND	ND	ND	ND	+	ND	+	+	+	+	ND	ND	ND	ND	ND	ND	+	ND	+	ND

LT1097d	ND	ND	ND	ND	ND	ND	+	+	+	ND	ND	+	ND	+	ND	ND	ND	ND	ND	ND	ND	ND
LT1097g	ND	ND	ND	ND	ND	ND	+	+	+	ND	ND	+	ND	+	ND	ND	ND	ND	ND	ND	ND	ND
LT1097h	ND	ND	ND	ND	ND	ND	+	+	+	ND	ND	+	ND	+	ND	ND	ND	ND	ND	ND	ND	ND
LT1099e	ND	ND	ND	ND	ND	ND	+	ND	ND	ND	+	+	+	ND	ND	ND	+	ND	+	+	+	ND
LT1099f	ND	ND	ND	ND	ND	ND	+	ND	ND	ND	ND	+	ND	ND	ND	ND	ND	ND	ND	ND	+	ND
LT1099g	ND	ND	ND	ND	ND	ND	+	+	+	ND	ND	+	ND	+	ND	ND	ND	ND	ND	ND	ND	ND
LT1099h	ND	ND	ND	ND	ND	ND	+	+	+	ND	ND	+	ND	+	ND	ND	ND	ND	ND	ND	ND	ND
LT1131c	ND	ND	ND	ND	+	ND	ND	ND	ND	ND	ND	+	ND	ND	ND	ND	ND	ND	ND	ND	+	ND
LT1131d	ND	ND	ND	ND	ND	ND	ND	ND	ND	ND	ND	ND	ND	ND	ND	ND	ND	ND	ND	ND	ND	ND
LT1131e	ND	ND	ND	ND	ND	ND	ND	ND	ND	ND	ND	ND	ND	ND	ND	ND	ND	ND	ND	ND	ND	ND
LT1131f	ND	ND	ND	ND	ND	ND	ND	ND	ND	ND	ND	ND	ND	ND	ND	ND	ND	ND	ND	ND	ND	ND
LT1131g	ND	ND	ND	ND	ND	ND	ND	ND	ND	ND	ND	ND	ND	ND	ND	ND	ND	ND	ND	ND	ND	ND
LT1132b	ND	ND	ND	ND	ND	ND	+	ND	ND	ND	ND	+	ND	ND	ND	ND	ND	ND	ND	ND	+	ND
LT1132c	ND	ND	ND	ND	ND	ND	+	ND	ND	ND	ND	+	ND	ND	ND	ND	ND	ND	ND	ND	+	ND
LT1132d	ND	ND	ND	ND	ND	ND	+	ND	ND	ND	ND	+	ND	ND	ND	ND	ND	ND	ND	ND	+	ND
LT1132e	ND	ND	ND	ND	ND	ND	+	ND	ND	ND	ND	+	ND	ND	ND	ND	ND	ND	ND	ND	+	ND
LT1132f	ND	ND	ND	ND	ND	ND	+	ND	ND	ND	ND	+	ND	ND	ND	ND	ND	ND	ND	ND	+	ND
LT1132g	ND	ND	ND	ND	ND	ND	+	ND	+	ND	ND	+	ND	ND	ND	ND	ND	ND	ND	ND	+	ND
LT1132h	ND	ND	ND	ND	ND	ND	+	ND	ND	ND	ND	+	ND	ND	ND	ND	ND	ND	ND	ND	+	ND
LT1143c	ND	ND	ND	ND	ND	ND	ND	ND	ND	ND	ND	ND	ND	ND	ND	ND	ND	ND	ND	ND	ND	ND
LT1143e	ND	ND	ND	ND	ND	ND	ND	ND	ND	ND	ND	ND	ND	ND	ND	ND	ND	ND	ND	ND	ND	ND
LT1143f	ND	ND	ND	ND	ND	ND	ND	ND	ND	ND	ND	ND	ND	ND	ND	ND	ND	ND	ND	ND	ND	ND
LT1143g	ND	ND	ND	ND	ND	ND	ND	ND	ND	ND	ND	ND	ND	ND	ND	ND	ND	ND	ND	ND	ND	ND
LT117+g	+	+	+	+	+	+	+	ND	+	+	+	+	ND	ND	+	+	ND	+	+	ND	+	ND
LT1173c	+	+	+	+	+	+	+	ND	+	+	+	+	ND	ND	+	+	ND	+	+	ND	+	ND
LT1173d1	+	+	+	+	+	+	+	ND	+	+	+	+	ND	ND	+	+	ND	+	+	ND	+	ND
LT1173e	+	+	+	+	+	+	+	ND	+	+	+	+	ND	ND	+	+	ND	+	+	ND	+	ND
LT1173f	+	+	+	+	+	+	+	ND	+	+	+	+	ND	ND	+	+	ND	+	+	ND	+	ND
LT1173g	+	+	+	+	+	ND	+	ND	+	+	+	+	ND	ND	+	+	ND	+	+	ND	+	ND

LT1173h	+	+	+	+	+	+	+	ND	+	+	+	+	ND	ND	+	+	ND	+	+	ND	+	ND
LT8703a	+	+	+	+	+	+	ND	ND	+	+	+	+	ND	ND	+	+	ND	+	+	ND	+	ND
LT8052a	+	ND	+	ND	+	ND	+	ND	+	+	ND	+	ND	ND	ND	+	ND	ND	ND	+	ND	+
LT8062a	+	+	+	+	+	ND	+	ND	+	+	ND	+	ND	ND	+	+	ND	ND	ND	+	ND	ND
LT8+25a	+	+	+	+	+	ND	ND	ND	+	ND	ND	+	ND	ND	ND	ND	ND	ND	ND	+	+	ND
LT8+79a	+	ND	ND	ND	ND	ND	+	ND	+	+	+	+	ND	ND	ND	ND	ND	ND	+	ND	+	ND
LT8+98a	+	ND	ND	ND	ND	ND	+	ND	+	+	+	+	+	ND	ND	ND	ND	ND	ND	ND	+	ND
LT823+a	+	ND	ND	ND	ND	ND	+	ND	+	+	+	+	ND	ND	ND	ND	ND	ND	+	ND	+	ND
LT8242a	+	ND	ND	ND	ND	ND	+	ND	+	+	+	+	ND	ND	ND	ND	ND	ND	+	ND	+	ND
LT8320a	ND	ND	ND	ND	ND	ND	+	+	+	ND	ND	+	ND	+	ND	ND	ND	ND	ND	ND	ND	ND
LT8371a	ND	ND	ND	ND	ND	ND	ND	ND	ND	ND	ND	+	ND	ND	ND	ND	ND	ND	ND	ND	ND	ND
LT8455a	ND	ND	+	ND	ND	ND	ND	ND	ND	ND	ND	+	ND	ND	ND	ND	ND	ND	ND	ND	ND	+
LT1097c	ND	ND	ND	ND	ND	ND	+	+	+	ND	ND	+	ND	+	ND	ND	ND	ND	ND	ND	ND	ND

* ND: Not detected.

Table S4 Results of antimicrobial susceptibility testing of 125 *E. coli* isolates from 11 households

Antimicrobial		Number of isolates (n)	Proportion (95%CI)
Ampicillin	R	122	97.6% (95 - 100%)
Cephalexin	R	117	93.6% (89 - 98%)
Cefpodoxime	R	125	100% (100 - 100%)
Ceftriaxone	M	1	0.8% (0 - 2%)
	R	123	98.4% (96 - 100%)
Mecillinam	R	4	3.2% (0 - 6%)
Ertapenem	M	18	14.4% (8 - 21%)
	R	0	0% (0 – 0%)
Augmentin	R	42	33.6% (25 - 42%)
Cefoxitin	R	32	25.6% (18 - 33%)
Gentamicin	R	38	30.4% (22 - 38%)
Amikacin	R	0	0% (0 – 0%)
Norfloxacillin	M	5	4.0% (1 - 7%)
	R	76	60.8% (52 - 69%)
Trimethoprim	M	1	0.8% (0 - 2%)
	R	96	76.8% (69 - 84%)
Nitrofurantoin	R	14	11.2% (6 - 17%)
Fosfomycin	R	6	4.8% (1 - 9%)

M: moderate susceptibility; R: resistant (see Table S9 for respective zone diameters and disk concentrations)

Table S5 Antimicrobial resistance genes in 125 *E. coli* isolates from 11 households

Resistance gene type	Antimicrobial resistance class	Isolates with resistance genes	
		n/Total isolates	Proportion (95%CI)
aac-type	Aminoglycoside and/or fluoroquinolone	67/125	53.6% (45 - 62%)
aad-type	Aminoglycoside	86/125	68.8% (61 - 77%)
str-type	Aminoglycoside	57/125	45.6% (37 - 54%)
*bla*ESBL-type	Beta-lactam (incl. 3rd & 4th generation cephalosporins)	109/125	87.2% (81 - 93%)
*bla*AmpC-type	Beta-lactam (incl. 3rd & 4th generation cephalosporins)	18/125	14.4% (8 - 21%)
*bla*OXA-type (non-ESBL)	Beta-lactam	49/125	39.2% (31 - 48%)
*bla*TEM-type (non-ESBL)	Beta-lactam	38/125	30.4% (22 - 38%)
dfrA-type	Trimethoprim	97/125	77.6% (70 - 85%)
sul-type	Sulfonamide	104/125	83.2% (77 - 90%)
qnr-type	Fluoroquinolone	8/125	6.4% (2 - 11%)
mph-type	Macrolide	92/125	73.6% (66 - 81%)
tet-type	Tetracycline	76/125	60.8% (52 - 69%)
catB-type	Phenicol	43/125	34.4% (26 - 43%)

Table S6 Summary of reference genomes used for intra-household core SNP comparison of 125 *E. coli* isolates from 11 households (using Snippy 3.0)

Household	Reference Isolate ID	Read depth	GC content (%)	Assembled genome size (bp)	Assembled contigs (n)	CDS (n)
HH08	LT1003c	94	50.7	5434577	553	5001
HH15	LT1030h2	61	50.0	5168471	249	4749
HH16	LT1029f	133	49.9	5245042	312	4904
HH24	LT8179a	81	51.1	5160657	178	4808
HH26	LT1044g	118	50.3	4985966	219	4653
HH39	LT1090f	113	50.8	5188047	305	4888
HH40	LT1082f	107	50.3	5164692	181	4840
HH48	LT1097g	129	51.1	5151025	339	4709
HH64	LT8371a	120	50.2	5153473	338	4694
HH65	LT1132e	122	50.0	5118820	288	4715
HH086	LT1173e	199	50.1	5141055	298	4744

bp: base-pairs

CDS: coding sequences

Read depth expressed as fold-coverage: each base sequenced on average the depth number of times

Paired-end sequence reads from each *E. coli* isolate in these 11 households was compared to a reference selected from that household as shown in **Figure 3** and Supplementary Figure 1.

Table S7. SNP distances matrix for *E. coli* ST69

	LT1003c	LT1003d	LT1003f	LT1003g	LT1003h	LT8062a	LT1132b	LT1132c	LT1132d	LT1132e	LT1132f	LT1132g	LT1132h	LT8455a
**LT1003c**	0	3	77999	6	4	24	4009	4009	4008	4009	4007	4007	4017	4006
**LT1003d**	3	0	78000	3	3	21	4008	4006	4005	4006	4004	4006	4016	4003
**LT1003f**	77999	78000	0	78003	77999	78015	77476	77474	77473	77474	77474	77470	77470	77471
**LT1003g**	6	3	78003	0	6	24	4011	4009	4008	4009	4007	4009	4019	4006
**LT1003h**	4	3	77999	6	0	24	4007	4007	4006	4007	4007	4005	4017	4004
**LT8062a**	24	21	78015	24	24	0	4029	4027	4026	4027	4025	4027	4037	4024
**LT1132b**	4009	4008	77476	4011	4007	4029	0	4	3	4	8	8	24	5
**LT1132c**	4009	4006	77474	4009	4007	4027	4	0	1	2	6	6	22	3
**LT1132d**	4008	4005	77473	4008	4006	4026	3	1	0	1	5	5	21	2
**LT1132e**	4009	4006	77474	4009	4007	4027	4	2	1	0	4	6	20	3
**LT1132f**	4007	4004	77474	4007	4007	4025	8	6	5	4	0	6	20	3
**LT1132g**	4007	4006	77470	4009	4005	4027	8	6	5	6	6	0	20	3
**LT1132h**	4017	40+6	77470	4019	4017	4037	24	22	21	20	20	20	0	19
**LT8455a**	4006	4003	77471	4006	4004	4024	5	3	2	3	3	3	19	0

Table S8. SNP distances matrix for *E. coli* ST963

	**LT8371a**	**LT8052a**	**LT1030c**	**LT1030d**	**LT1030e**	**LT1030f**	**LT1030g1**	**LT1030h2**
**LT8371a**	0	35	82	68	84	107	101	106
**LT8052a**	35	0	51	65	55	78	70	75
**LT1030c**	82	51	0	22	12	35	27	32
**LT1030d**	68	65	22	0	26	49	41	46
**LT1030e**	84	55	12	26	0	35	31	32
**LT1030f**	107	78	35	49	35	0	50	51
**LT1030g1**	101	70	27	41	31	50	0	49
**LT1030h2**	106	75	32	46	32	51	49	0

Table S9 Antimicrobial zone diameters used in Kirby-Bauer disk diffusion phenotypic assays

**Antimicrobial**		**Susceptible zone size (mm) ≥**	**Resistant zone size (mm) <**
Ampicillin (10µg)	AMP10	14	14
Cephalexin (30µg)	LEX30	14*	14*
Cefpodoxime (10µg)	CPD10	21*	21*
Ceftriaxone (30µg)	CRO30	25	22
Mecillinam (10µg)	MEC10	15	15
Ertapenem (10µg)	ETP10	25	22
Augmentin (amoxicillin 20µg + clavulanic acid 10µg)	AUG30	16*	16*
Cefoxitin (30µg)	FOX30	19†	19†
Gentamicin (10µg)	GM10	17	14
Amikacin (30µg)	AK30	18	15
Norfloxacillin (10µg)	NOR10	22	19
Trimethoprim (5µg)	TMP5	18*	15*
Nitrofurantoin (100µg)	NI100	11*‡	11*‡
Fosfomycin (200µg)	FOT200	24*‡	24*‡

* Clinical breakpoint for uncomplicated UTI

† Screening breakpoint for ACBL production

‡ *E. coli* only

ESBL confirmatory testing was performed using three paired disk tests [D62C ESBL cefotaxime Paired ID disks (Mast group, Bootle, U.K.); D63C ESBL cefepime Paired ID disks (Mast group); D64C ESBL ceftazidime Paired ID disks (Mast group)]. Urine isolates were tested for ESBL-producing phenotype using two paired disk tests [D62C ESBL cefotaxime Paired ID disks (Mast group); D64C ESBL ceftazidime Paired ID disks (Mast group)].

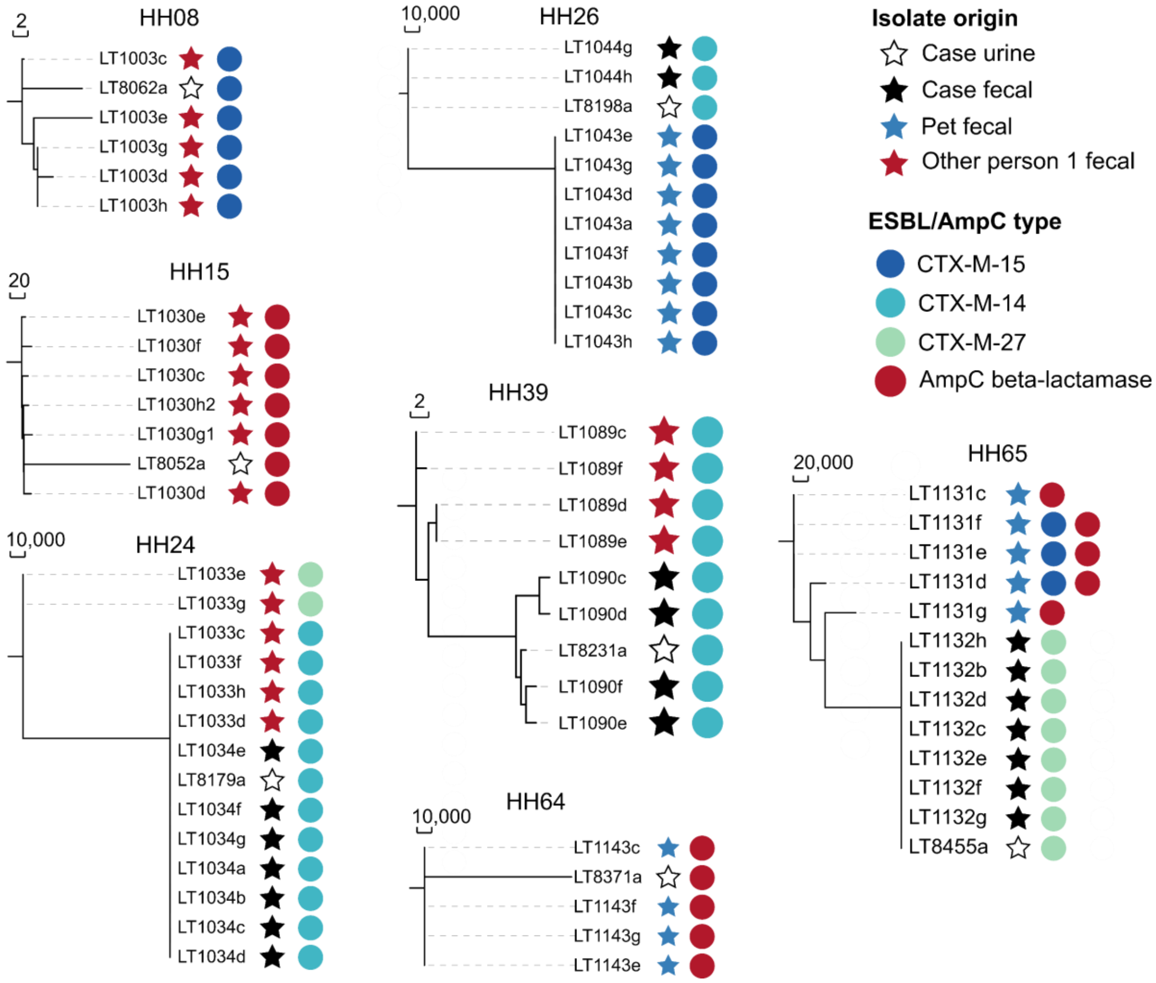

Figure S1. Neighbour joining core SNP phylogenies, generated using Snippy [1], for ESBL- and/or ACBL-producing *E. coli* isolates from seven households. The scale bar represents the branch length in number of SNPs.

[1] Seemann T. Snippy: Fast Bacterial Variant Calling from NGS Reads, 2015. https://github.com/tseemann/snippy.
